# The effects of a temporal framing manipulation on environmentalism: A replication and extension

**DOI:** 10.1371/journal.pone.0246058

**Published:** 2021-02-11

**Authors:** Samantha K. Stanley, Anna Klas, Edward J. R. Clarke, Iain Walker

**Affiliations:** 1 Centre for Applied Psychology, University of Canberra, Canberra, Australian Capital Territory, Australia; 2 Research School of Psychology, Australian National University, Canberra, Australian Capital Territory, Australia; 3 Misinformation Lab, School of Psychology, Deakin University, Geelong, Victoria, Australia; 4 School of Science, Psychology and Sport, Federation University Australia, Berwick, Victoria, Australia; Texas Tech University, UNITED STATES

## Abstract

Recent research promotes comparing the current state of the environment with the past (and not the future) to increase the pro-environmental attitudes of those on the political right. We aimed to replicate this temporal framing effect and extend on research in this area by testing the potential drivers of the effect. Across two large-scale replication studies, we found limited evidence that past comparisons (relative to future comparisons) increase pro-environmentalism among those with a more conservative political ideology, thus precluding a full investigation into the mediators of the effect. Where the effect was present, it was not consistent across studies. In Study One, conservatives reported greater certainty that climate change was real after viewing past comparisons, as the environmental changes were perceived as more certain. However, in Study Two, the temporal framing condition interacted with political orientation to instead undermine the certainty about climate change among political *liberals* in the past-focused condition. Together, these studies present the first evidence of backfire from temporal frames, and do not support the efficacy of past comparisons for increasing conservatives’ environmentalism. We echo recent calls for open science principles, including preregistration and efforts to replicate existing work, and suggest the replication of other methods of inducing temporal comparisons.

## Introduction

Climate change is a politically polarized issue, with liberals often supporting climate action and conservatives more likely to deny the existence of climate change [1]. This is particularly the case in Anglosphere nations, including the United States, Australia and the United Kingdom [2, 3]. The conservative movement against climate science has damaged climate change mitigation efforts by undermining the perceived strength of the evidence for climate change, as shown by the United States failing to meet obligations under the Kyoto Protocol [4] and its more recent withdrawal from the Paris agreement [5, 6].

A recent meta-analysis demonstrated that conservative ideology and right-wing votership are stronger predictors of climate change denial than other demographic correlates such as gender, age, and education [1]. This means that the denial of climate change is potentially driven by stable ideological factors, thus explaining the ineffectiveness of climate change communication strategies that rely on merely conveying the scientific information [7, 8]. Climate change communication strategies must overcome ideological barriers, not information deficits, to motivate action.

One proposed strategy to increase the effectiveness of climate change communication for conservatives has been to employ message frames that align with their apparent preference for the past. Previous research has shown that message frames that use a past-focused frame (for example, by comparing the current state of the environment to how it used to be) rather than a future-focused frame (comparing the current state of the environment to how it will be in the future), eliminates the negative effect of conservatism on pro-environmental attitudes [9]. Our research aims to replicate and extend on literature that positions the temporal framing of environmental issues as a way to bridge the ideological divide between conservatives’ and liberals’ environmental attitudes in several important ways. Firstly, explanations for the effect are limited and were not tested in the original research. Secondly, it is unclear if the temporal framing effect observed on pro-environmental attitudes extends to motivate more meaningful forms of environmentalism, such as increasing pro-environmental action, support for climate policy, or acceptance of climate change. Finally, the effect has yet to be replicated with a multidimensional representation of political ideology. Our program of research aims to address these limitations.

### Conservative denial of climate change

Political affiliation is an important predictor of environmental attitudes [1]. When compared to their left-wing or liberal counterparts, those with a right-wing or conservative political orientation are typically more skeptical of climate change science [10, 11], hold less positive pro-environmental attitudes [12], and are more opposed to climate change policy [13]. Given this, much focus has been on understanding the mechanisms that drive conservatives’ denial of climate change and climate inaction.

One likely reason why climate change is politically polarised is that from the 1990s, conservative think tanks responded to the environmentalist movement with a series of messages that sought to undermine action on climate change [14]. These messages attempted to shroud the scientific basis of climate change in doubt, emphasized the harm that mitigating action would cause to the economy or national security, and claimed that on balance, the effects of climate change would be positive (e.g. benefitting agriculture, human quality of life and health). Over 90% of books that refute climate change have since been linked to conservative think tanks [15, see also 16], and climate change counter-movement organisations appear to draw the majority of their financial support from conservative foundations [17]. Oreskes and Conway [18] suggest that conservative opposition to international agreements on climate change is also driven by the perception that such action threatens the free market economic system.

Climate change communications have shown some success in motivating belief and action on climate change; however, they are not equally persuasive to members of different groups, thus suggesting a tailored approach (i.e. segmentation [19, 20]). Potentially contributing to the ideological divide on climate change, most communication strategies tend to frame climate change in ways that appeal to liberals and their values. For instance, existing appeals have found greater success motivating those holding a liberal political ideology through highlighting action on climate change as reflecting values of harm and care [19], necessary for the future [9], or by describing those at risk of the effects of climate change [21]. These messages each construct climate change as relevant to liberal values, such as environmental preservation or equity. Further still, these same messages have either been ineffective for conservatives or have amplified conservatives’ doubt regarding climate change, thus exerting the opposite intended effect [21]. Together, these studies provide support for taking a segmented approach to communications, which involves developing and implementing messages that align with certain political ideologies, in order to achieve broad-based political support for climate action.

### Promoting (or *de*motivating) conservative engagement with climate change

Currently, some evidence suggests conservatives are sensitive to subtle changes in the framing of climate change. For example, denial is higher among conservatives when the issue is referred to as ‘global warming’ rather than ‘climate change’, while liberals are concerned about the issue regardless of wording [22, though see 23]. Liberals and conservatives also differ on dimensions such as personality and values [24], and moral foundations [25], which have been leveraged in communication campaigns to increase conservative support for climate action.

Analyses of the content of conservative messages refuting climate change suggest that this group is particularly sensitive to threats to the socioeconomic system [14]. With this knowledge, Feygina, Jost and Goldsmith [26] developed a message that framed pro-environmental behaviour as preserving the current way of life. This was effective in increasing pro-environmental intentions among those high in system justification (one pillar of conservative political ideology [27]). Consistent with the sensitivity towards protecting the economic system, conservatives are less supportive of a pro-environmental society when this is described at the expense of the economy [28]. Given that conservatives may favour economic concerns, it is logical to assume that framing climate change inaction as costly might skew decisions in favour of the environment. However, while Clayton, Koehn and Grover [29] replicated the main effect of political ideology on environmental attitudes, their frame emphasizing the economic harm associated with environmental disaster in the context of an oil spill was ineffective. Furthermore, emphasizing the threat to national security associated with climate change caused a backfire effect among those dismissive or doubtful about the issue, instead making them feel more anger and less hope [30]. Broadly speaking, a backfire effect is when a climate message intended to increase a specific positive climate outcome results in the opposite effect, and this is more likely to occur when information presented in a message challenges an individual’s pre-existing political beliefs [21]. Further supporting this, another study found that four separate positive frames urging climate policy action (including national security and economic opportunity frames) did not increase belief in anthropogenic climate change [31]. Therefore, while conservatives might be particularly sensitive to threats to the system, including economic and security threats, there is little evidence that framing environmental messages to align with these threats increases environmentalism.

Environmental messages have also been tailored to target moral preferences held more strongly by conservatives. People across the political spectrum typically hold varying moral preferences: liberals value individualizing foundations of harm, care, and fairness, and conservatives prefer binding foundations such as respect and ingroup loyalty [32, 33, but see 34]. Consistent with these preferences, Kidwell et al. [35] have shown that an appeal based on binding moral foundations increases conservatives’ recycling intentions and behaviour, and is rated as clearer and more credible than a message based on individualizing foundations. Wolsko et al. [36] similarly demonstrated that environmental appeals that reference purity, obeying authority, or environmentalism as an act of patriotism, which can be broadly categorized as aligning with binding moral foundations, show promise in increasing conservatives’ environmental conservation intentions. Importantly, these findings show that targeting a message that resonates with conservative values may also backfire for liberals.

Related to concerns about purity, conservatives are especially sensitive to disgust triggered by contamination concerns [37]. However, while some framing studies demonstrate that health frames elicit hope [30], or increase pro-environmental attitudes (though not action [38]), those studies that consider the interaction between frames and political ideology paint a more complicated picture. Health frames can backfire to decrease climate policy support among conservatives [21], are rated as more biased by those who are dismissive of climate change [39], and may even backfire for liberals [40]. Although health frames may be rated overall more positively than messages emphasizing the economic, environmental, moral, or national security implications of climate change, Feldman and Hart [41] noted that Republicans do not show this preference. Taken together, current attempts to promote action by appealing to conservative values have returned mixed results.

Instead of leveraging conservative values in message frames, researchers have turned to the conservative preference for the past. Baldwin and Lammers [9] suggest that the temporal focus of many environmental campaigns does not align with conservatives’ preference for the past. Importantly, while environmental campaigns highlight the future consequences that might be encountered—or avoided—depending on our actions, this focus on the future might lead conservatives to disengage from climate change communication specifically. Supporting this, Baldwin and Lammers [9] demonstrated that conservatives rate messages that compare how the environment is now to how it was in the past more favourably than those comparing the current state of the environment to its projected state in the future (Study 1–3). The authors suggest that conservatives have this preference because they hold “a resistance to progressive change” (p. 14956). This preference for the past might explain why conservatives give more to charities that are past-focused than future-focused (Study 4b & 5–6), and why existing climate change communications, which are more often future-focused (Study 4a), are less successful at motivating conservatives to act on climate change.

Providing support for the generality of the temporal framing effects, Lammers and Baldwin [42] recently showed that similar past comparisons broaden to build conservative support for other issues typically supported by liberals. Presenting conservatives with idealised messages comparing the present to the past increased support for issues such as gun control legislation, social diversity, immigration, and leniency on crime. Conservatives were less supportive of the same issues when messages compared the present to the future. Across these applications of the temporal comparison manipulation, Baldwin and Lammers [9] and Lammers and Baldwin [42] found very little evidence that the past-focused messages backfire among liberals, and therefore past comparisons show promise in uniting attitudes on important issues.

### Explanations for the temporal framing effect

While the research to date on temporal comparisons shows promise in motivating environmentalism, explanations for the effect are limited and were not comprehensively tested in the original research. Lammers and Baldwin [42] speculated that conservatives prefer past comparisons because they are more prone to experiences of nostalgia. In one study, they manipulated temporal focus by presenting a message about social diversity within a vintage comic (past-focused) versus a modern comic. Feelings of nostalgia explained conservatives’ greater acceptance of a social diversity message from a vintage comic (compared to a modern-style comic; Study 5). They also showed that conservative support for social justice appeals as people “getting what they deserve” (which we note could instead be interpreted as meritocracy or system justification, principles that conservatives are known to prefer [27]) is higher with a past-focus, and this effect is driven by conservatives’ greater dispositional nostalgia (Study 6). Together, this research hints at the role of nostalgia underlying conservatives’ receptiveness to past-focused messages, as both nostalgia invoked from a message (state nostalgia, Study 5) and stable individual differences in nostalgia (trait nostalgia, Study 6) help to explain the association between political orientation and support for liberal issues.

This potential explanation for the temporal framing effect, and its impact on conservatives, was not tested in the series of experiments reported in Baldwin and Lammers [9] on motivating pro-environmentalism. Furthermore, Lammers and Baldwin [42] suggest a “positive evaluation of the past” (p. 600) is a core pillar of conservative ideology. This seems reasonable, but has not been empirically tested. A positive evaluation of the past implies that conservatives may tend towards endorsing a past-positive time perspective [43], which is a general construct capturing the extent one thinks about the past, and the valence of these thoughts. No time perspective measure was included in the temporal framing research, though previous nostalgia measures have been developed from the past positive dimension of time perspective (e.g. [44]), and there are robust positive associations between *future* time perspective and greater pro-environmentalism [45]. Therefore, the association between political ideology and perceptions of time ought to be explored further, especially if the political polarization of climate change is attributed to a fundamental difference in temporal focus.

Another potential explanation for the temporal framing effect is that conservatives are less convinced by future-focused messages because these are characterised by greater uncertainty (what might happen in the future) than past-focused messages (what has already happened). Baldwin and Lammers [9] first raised—and dismissed—the possibility that the temporal framing effect is driven by a type of perceived uncertainty. They operationalised uncertainty in a general way: their participants rated the extent they experienced *feelings of* uncertainty (e.g. feeling restless, unsure) after viewing the temporal comparison images. These general feelings of uncertainty did not eliminate the effect of the temporal comparison manipulation. However, they did find a marginally significant interaction between feelings of uncertainty and political orientation, with conservatives reporting greater feelings of uncertainty than liberals in the future-focused condition. They also measured the need for cognitive closure, which is a motivated tendency to avoid confusion and ambiguity [46]. Although conservatives scored higher in this construct, it did not interact with the temporal framing condition to influence environmentalism.

While these analyses were taken as evidence that the temporal framing effect was not explained by differences between felt uncertainty in past- and future-focused conditions, these uncertainty constructs are a step removed from (un)certainty about the environmental changes themselves. Specifically, Baldwin and Lammers’ measures refer to vague ‘feelings’ of uncertainty that are not explicitly directed towards the image rating task, and stable individual differences in need for certainty. However, it is possible that the past-comparisons themselves are viewed as more certain (this has already happened) than future-comparisons (this might happen in future), independent of general feelings of uncertainty. If it is the case that past comparisons are viewed as representing more certain environmental changes than future comparisons, then this may explain why conservatives, who are more sensitive to the need for certainty, respond more favourably to past comparisons.

Those who are less certain about several aspects of climate change, including whether it is happening, how serious it will be, and the extent of scientific agreement on the issue, are less concerned about the issue and have lower intentions to act in pro-environmental ways [47]. These are aspects of psychological distance. Another dimension of psychological distance is temporal distance: those who believe we will not feel the effects of climate change until further in the future show the same pattern of disengagement. Changes that have already occurred are likely more psychologically ‘close’ along temporal and certainty distance dimensions than changes that may occur in the future. It is possible that the past comparisons increase environmentalism through decreasing the perceived temporal distance of climate change. Previous research manipulating psychological distance is inconsistent though [48], so while this explanation is less convincing, it warrants testing.

Our research aims to explore the potential mechanisms underlying the temporal framing effects, and to extend the work conducted to date. We also aim to test what forms of environmentalism shift in response to temporal frames. Baldwin and Lammers’ [9] third study used the new environmental paradigm (NEP [49]), as the only environmental indicator. The NEP has been criticised for lacking construct validity—it could be capturing a general environmental worldview, attitudes, values, or concern, and indeed has been used as a proxy for all of these things [50]. Other framing research has demonstrated that while frames might increase support for some aspects of environmentalism, they might simultaneously decrease support for other environmental indicators. In particular, Levine and Kline [38] observed a simultaneous increase in policy support and decrease in activism intentions in response to their climate change frame appealing for action to improve one’s own material wellbeing. It is therefore important to examine whether the temporal framing effect affects other forms of environmentalism, including support for climate change policy, given that the original aim of the conservative countermovement was to undermine support for environmental policy [4].

We also note that Baldwin and Lammers [9] indexed political ideology along a single left-right dimension. This standard approach to measuring political ideology is potentially problematic, as individuals may be unwilling to identify with either “liberal” or “conservative” labels due to perceived negative connotations of the terms [51], or a lack of understanding of what the labels refer to [52], or because some may not equate ‘liberal’ with ‘left-wing’ and ‘conservative’ with ‘right-wing’. Furthermore, a single dimension does not represent those whose political views are compiles (e.g., those who are socially conservative but economically liberal [53, 54]). This has prompted other researchers to attempt to understand the drivers of conservative climate denial by first considering the origins of political ideology, and its multidimensional nature.

Views on both the environment [55] and politics [27] are predicated on more foundational beliefs about how the social world ought to be structured (in a strict hierarchy, versus egalitarian) and controlled (strong, tough governance, versus individual freedom). These ideological stances are indexed by social dominance orientation (SDO [56]), the relative tolerance of inequality and intergroup dominance, and right-wing authoritarianism (RWA [57]), which refers to a tendency to submit to authorities, commit to norms and traditions, and a preference for strict, punitive leadership. Although both RWA and SDO constructs were originally conceptualised as personality traits, more recent literature considers them to be measures of ideological belief systems [27], which can be used to index the underlying dimensions of political ideology. Those oriented towards social dominance and authoritarianism typically deny climate change at similar rates [58] and are generally less willing to make sacrifices in their personal lives for the benefit of the environment [59, 60].

Recent advances in the measurement of both social dominance and authoritarianism delineate them into multidimensional constructs. Anti-egalitarianism (or SDO-E) is the rejection of group-based equality, and support for dominance (SDO-D) entails a preference for high-status groups’ active oppression over subordinate groups [61]. In the environmental sphere, endorsement of SDO-E is associated with the rejection of climate science and an unwillingness to make personal sacrifices for the environment, while SDO-D is a weak or inconsistent predictor [62, 63]. The dimensions of RWA include favouring assertive, punitive social control (referred to in the literature as *Authoritarianism* or *Authoritarian Aggression*), submitting to authority (labelled *Conservatism* or *Submission*), and traditional values and norms (*Traditionalism* or *Conventionalism*). Reese [64] found that the preference for tradition and authoritarian aggression were associated with lower pro-environmentalism, while willingness to submit to authority predicted increased pro-environmentalism. Clarke et al. [62] similarly showed that those who favoured traditionalism were more likely to deny climate change in many forms, through greater rejection of the science, existence, impacts, and anthropogenic nature of climate change.

Taken together, limited evidence therefore points to traditionalism as most consistently underlying the RWA-environmentalism link, and SDO-E as explaining why those who score relatively higher in SDO are less pro-environmental. Importantly, the patterns of associations in this work point to the utility of considering political ideology as an expression of SDO and RWA, and supports the separation of these constructs into two (for SDO) and three (for RWA) dimensions, respectively.

### Current studies

We aim to replicate and extend Baldwin and Lammers’ [9] third study, where participants were shown images of environmental changes that either have occurred (comparing past with present) or will occur (comparing present with future). We will do this using a larger sample, recruited from two countries where climate change is heavily politicized: the United States (Study One) and the United Kingdom (Study Two). In addition to the liberal-conservatism continuum used by Baldwin and Lammers, in Study One, we include SDO as one of the underlying dimensions of political conservatism to examine how the temporal comparison frames operate across individual differences in support for intergroup dominance (SDO-D) and inequality (SDO-E). We extend on this analysis of the foundational ideological attitudes underlying the temporal framing effect in Study Two by also including the dimensions of RWA. We first aim to replicate their main interaction between political ideology and temporal focus, and then extend this work by testing the possible explanations for these effects outlined above: perceived certainty, psychological distance, nostalgia, and past positive time perspective.

## Study one

In Study One, we aimed to replicate Baldwin and Lammers’ [9] temporal framing effect, expecting to find that this interacts with political orientation to influence environmentalism. We employed a 7-point political ideology scale so we could directly compare our results to those of Baldwin and Lammers, who also used a single item measure of political ideology. We expected to observe the same pattern of results as the original study, with liberals endorsing greater pro-environmentalism than conservatives (regardless of framing condition), and the past frame closing the gap between liberal and conservative responses. These analyses are repeated with the dimensions of SDO in place of political orientation, to see whether we could replicate and extend upon Baldwin and Lammer’s findings with a more nuanced measure of political orientation. We expected that individual differences in the anti-egalitarianism component would predict responses to the frame.

We also examine the possible mechanisms behind the temporal framing effect, and expected greatest support for our certainty explanation: that the reason conservatives are more responsive to the past frame is because the environmental changes are perceived as more certain in this condition than are the projected changes reported in the future-focused condition. Lastly, although previous research implies that conservatives may have a preference for the past, we empirically test this in Study One by examining the correlations between ideology and time perspective dimensions.

## Method

### Participants

Participants were recruited via Prolific and compensated US$1.83 for completing the ten-minute survey. Participation was limited to those living in the United States (consistent with [9]). As we aimed to replicate Baldwin and Lammers’ [9] finding, as well as extend on this research by examining what might underlie this effect (i.e. time perspective, nostalgia, psychological distance, certainty), we aimed to at least double the authors’ average sample size, increasing this by a buffer of 10% in case of participant withdrawal or missing data. To recruit a roughly even distribution of individuals across the political spectrum, we alternated between open recruitment and recruiting conservatives only, which returned a relatively normal distribution across the political orientation spectrum (*M* = 3.72, *SD* = 1.87 on a 7-point scale from very liberal to very conservative). The final sample consisted of 535 U.S. adults (51.6% male) between 18 and 73 years old (*M* = 32.61, *SD* = 12.17).

The University of Canberra Human Ethics Committee granted ethical approval for both Study One and Study Two, and in both cases participants consented to participating in the research by continuing on to the survey after reading an onscreen information sheet. Data for both Study One and Study Two are publicly available on the OSF: https://osf.io/uydgf/

### Materials and procedure

#### Part I

Participants were asked to locate their political views on a 7-point scale from 1 (very liberal) to 7 (very conservative). We also administered the 8-item SDO scale [61], which assessed agreement with SDO-E (e.g. “We should do what we can to equalize conditions for different groups”, α = .85) and SDO-D (e.g. “Some groups of people are simply inferior to other groups”, α = .78), which participants responded to on 7-point Likert scales.

Participants also completed Zhang, Howell and Bowerman’s [65] 15-item Shortened Zimbardo Time Perspective Inventory. We opted for this shortened version rather than the full (56-item) original version of the scale to avoid participant fatigue. This measure indexed present hedonistic (“I make decisions on the spur of the moment”, α = .72), present fatalistic (“Since whatever will be will be, it doesn’t really matter what I do”, α = .52), past negative (“I think about the bad things that have happened to me in the past”, α = .89), past positive (“I enjoy stories about how things used to be in the ‘good old times’”, α = .68), and future positive time perspective (“I complete projects on time by making steady progress”, α = .73) with three items each. The alphas of some of these ZTPI subscales was below the standard threshold for reliability, which is often the case with shortened scales, and may be a limitation of this work. Zimbardo’s work on time perspective does not include a future-negative perspective, so we included four items adapted from previous scales [66–69] that were acceptably reliable (α = .79, example item: “I am uncertain about my future”). For all time perspective items, participants indicated on a 5-point scale the extent to which the statement was very untrue to very true of them.

Participants also completed Baldwin, White and Sullivan’s [70] nostalgia measure (α = .82) by rating the extent to which they feel “nostalgic”, “sentimental” and “longing” from not at all (1) to a great deal (5). To get a similar estimate of the extent to which participants experienced future-directed emotions, participants used the same scale to rate the extent to which they were “excited for the future”, “looking forward to what comes next in life”, and “eagerly anticipating the future” (α = .92).

#### Part II

Participants were randomly allocated to either the past- or future-focused condition and shown a series of 14 photograph pairs used in Baldwin and Lammers [9]. In the past-focused condition, these showed a set of environmental changes where the more pristine environment was labelled ‘past’, and the degraded environment was labelled ‘present’. The same series of image pairs were used in the future-focused condition, labelled ‘present’ (pristine) and ‘future’ (degraded). To ensure that participants attended to the photographs, participants rated the extent to which they believed the changes depicted in each photograph set were caused by humans, using a sliding scale from 1 (entirely natural causes) to 100 (entirely human causes).

#### Part III

Participants were then presented with items designed to measure the remainder of our proposed mediators and the environmentalism measures.

*Perceived certainty*. We asked participants in the past-focused condition [and future-focused condition] “How certain are you that the changes you saw in the photographs have happened [will happen]?” on a sliding scale from 0 to 100.

*Psychological distance*. Spence et al.’s [47] item was used to measure the temporal distance of climate change. Participants were asked “When, if at all, do you think the United States will start feeling the effects of climate change?”, with response options from “we are already feeling the effects” (1) to “never” (7), such that higher scores are recorded as greater experiences of psychological distance.

*Climate change belief*. We used two related items (‘Climate change is real’ and ‘Climate change is caused by humans’, *r* = .75, *p* < .001) to index belief in climate change. We also asked participants to rate on a sliding scale how certain they were that climate change is happening (from 0 to 100) and, if they responded with anything above zero, what they think the main causes of changes in climate are, from entirely natural causes to entirely human causes.

*Willingness to sacrifice*. Liu and Sibley’s [71] 2-item willingness to make sacrifices for the environment scale measured willingness to make lifestyle and daily routine changes (*r* = .90, *p* < .001).

*Climate change policy support*. We also employed Bateman and O’Connor’s [13] climate change policy support scale, which gauges support for six mitigation strategies (e.g. “Cleaner energy sources such as wind and solar power, and other renewable sources”, α = .90) and five adaptation measures (e.g. “Managing tree species and forestry practices that are less vulnerable to storms and fires related to climate change”, α = .88).

*Behavioural measure*. To test whether the temporal framing effect extends to activism, we presented participants with two petitions they could choose to sign. Screenshots of two real petitions were taken from Change.org and presented to participants with the option of signing (see S1 Appendix). All details of the petitions were consistent (same picture used, edited to appear that each had the same number of signatures), but one advertised ‘Stop Gov’t HOAX Of Climate Change’, and the other ‘Acknowledge the Reality of Climate Change’. We asked participants which, if any, they would like to sign, as they would get the link to the petition in the debrief. However, the debrief instead explained that although both petitions they saw were real, they were now closed and no longer accepting signatures.

### Data analysis

We used the same data analytic strategy as Baldwin and Lammers [9]. Specifically, we began by regressing each environmental outcome measure on condition (past-focused versus future-focused) and political orientation (continuous measure from liberal to conservative) in one step, and in a second step including the interaction term (political orientation * condition). Where the interaction term was significant, this was followed up using the Johnson-Neyman technique (using the interactions package in R [72]) to determine at what point along the political orientation spectrum significant differences emerged in liberals and conservatives to each condition. These analyses were then repeated by including the two dimensions of SDO in place of political orientation. For the categorical dependent variable, which asked participants whether they would like to sign one of two petitions or no petition at all, we used multinomial logistic regression in SPSS. This analysis was appropriate for the petition variable, as the dependent variable was nominal, with mutually exclusive and exhaustive response options (sign petition: yes—stop hoax, yes—acknowledge reality, no).

For our main analyses with political orientation, we also tested assumptions of regression analysis. There were no major violations of linearity, normality or homoscedasticity for variables in the analyses. We did not detect any multivariate outliers (highest Mahalanobis’ distance was 7.66, Cook’s distance was .03). Multicollinearity was not an issue as the predictors (political orientation, condition) were independent, and this assumption does not apply when interaction terms are built within the regression to check for interaction effects.

We planned to follow up significant political orientation * condition interactions to test the proposed explanations for the effects: certainty, psychological distance, nostalgia, and past positive time perspective. This followed Lammers and Baldwin’s [42] approach of mediated moderation, using model 8 of the Process macro for SPSS [73] for mediators that may have been affected by the frame (certainty, temporal distance), and model 15 for those mediators that were thought to be more stable, and measured prior to the temporal framing manipulation (nostalgia, past-positive time perspective). Assumptions of normality, homoscedasticity and linearity were tested and met for all models.

## Results and discussion

### Replicating the temporal framing effect

Our first set of analyses, reported in Table 1, tested the efficacy of past- and future-focused messages for individuals across the political ideology spectrum. We regressed each environmental outcome variable separately onto condition (past versus future) and political orientation (continuous measure) in Step 1, and the interaction term (condition * political orientation) in Step 2.

**Table 1:** Standardized regression coefficients regressing each DV on political orientation, condition, and the interaction term.

	Climate change belief	Climate change certainty	Climate change causes	Willing to sacrifice	Support mitigation	Support adaptation
**Step 1**	R^2^ = .384***	R^2^ = .304***	R^2^ = .328***	R^2^ = .199***	R^2^ = .285***	R^2^ = .120***
Political orientation	-.618***	-.546***	.572***	-.443***	-.532***	-.341***
Condition	-.049	-.088*	-.008	-.065	-.053	-.065
**Step 2**	ΔR^2^ = .002	ΔR^2^ = .009**	ΔR^2^ = .000	ΔR^2^ = .000	ΔR^2^ = .002	ΔR^2^ = .001
Political orientation	-.499***	-.269*	.592***	-.388**	-.393**	-.269*
Condition	.031	.099	.005	-.028	.041	-.016
Political orientation X condition	-.148	-.344**	-.024	-.068	-.173	-.090

Note.

*** p < .001,

** p < .01,

* p < .05

Political orientation significantly predicted responses to all environmental outcome measures, with more conservative scorers giving less pro-environmental responses. Specifically, greater conservatism was associated with lower belief in climate change, less certainty that climate change was happening, higher likelihood of attributing climate change to natural causes, lower willingness to make sacrifices for the environment, and less support for mitigation and adaptation strategies.

**Fig 1:**
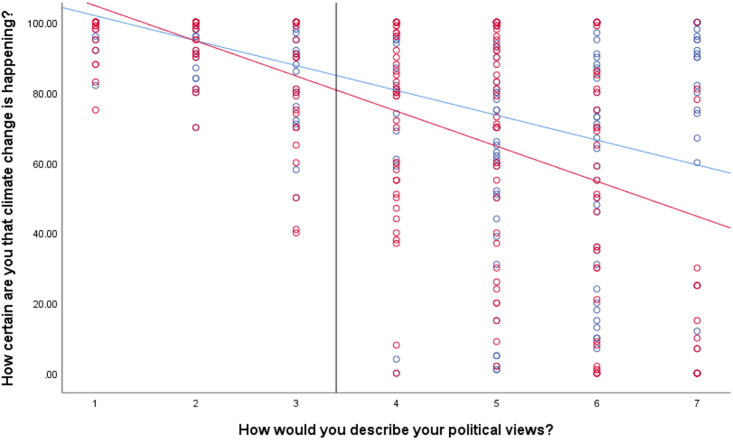
Association between political orientation and certainty that climate change is happening by past (blue) and future (red) temporal framing condition. Solid vertical line represents the Johnson-Neyman value. To the right of this, the differences in certainty ratings by condition are significant.

Temporal framing condition was only a significant predictor of responses to the certainty measure (those in the past-focused condition reported slightly higher certainty), and this was the only continuous outcome measure where we observed a significant political orientation * condition interaction. Although the interaction was significant, the effect was small (ΔR^2^ = .009). Following up this interaction using the Johnson-Neyman analysis indicated that the temporal framing effect emerged beyond a score of 3.40 on the liberal-conservative scale. That is, participants who placed their political orientation at the midpoint or on the conservative side of the scale (*N* = 280) were significantly (*p* < .05) more certain that climate change is happening in the past-focused condition than the future-focused condition (see Fig 1). For participants identifying as liberal, their degree of certainty that climate change is happening is about the same, regardless of framing condition. Although conservatives are more certain in the past condition, they are still on average less certain than liberals in either condition.

We used multinomial logistic regression to compare the likelihood of agreeing to sign the pro-climate petition (coded as 1; *N* = 219), hoax petition (-1; *N* = 15), or neither petition (0, reference category; *N* = 301). We built a custom model so that the interaction term (condition*political orientation) could be included along with framing condition (categorical, entered as a factor) and political orientation (continuous, entered as a covariate). This model provided poor fit to the data (Pearson chi-square statistic = 32.92, *p* = .034), however showed promise, as the predictors explained the outcome variable better than an intercept-only model (χ^2^(6) = 99.57, *p* < .001). The political orientation * condition interaction (χ^2^(2) = 2.66, *p* = .264) was not significant, potentially driving the poor fit. Indeed, removing the interaction term improved fit to an acceptable level (Pearson chi-square statistic = 33.19, *p* = .059; χ^2^ (4) = 96.91, *p* < .001). Consistent with the findings described above, framing condition was not a significant predictor (χ^2^(2) = 2.60, *p* = .272) of responses to the petition item, while political orientation was (χ^2^(2) = 94.28, *p* < .001). This analysis indicated that conservative individuals were significantly less likely to agree to sign the pro-climate petition than no petition (B = -.48, *p* < .001), however they were no more likely to sign the hoax petition than no petition (B = .28, *p* = .096). Each step up the scale on conservatism decreased the likelihood of signing the pro-climate petition by just over a third (Odds ratio = 0.62).

### Potential explanations of the temporal framing effect

Our next step was to test whether the temporal framing effect observed on the climate change certainty item could be explained by any of our four explanatory variables: 1. certainty that the changes had happened (or will happen), 2. temporal psychological distance, 3. nostalgia, and 4. past-positive time perspective.

**Fig 2:**
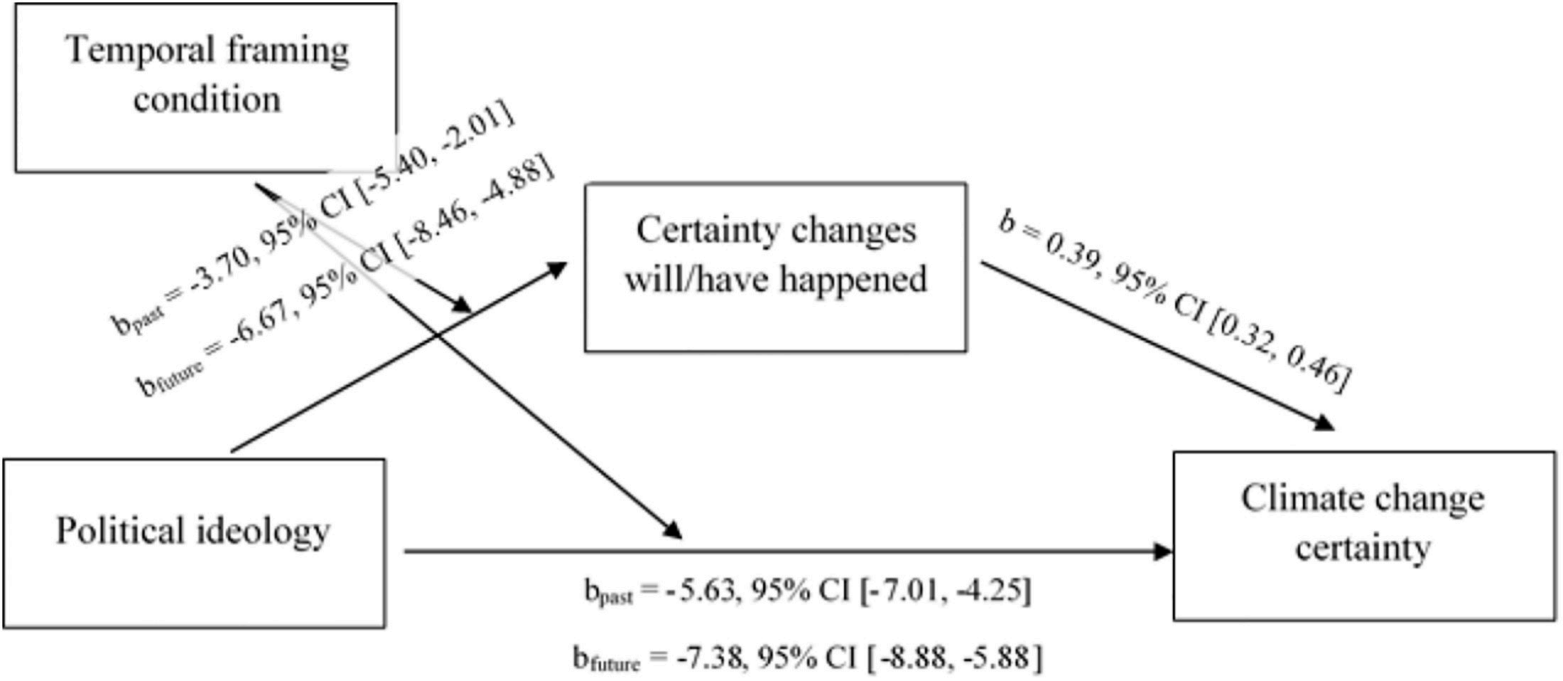
Effect of political ideology on climate change certainty is mediated by certainty that environmental changes have/will happen, dependent on temporal framing condition.

We began by testing the two potential mediators that may have been affected by the temporal frame (certainty and temporal distance) using Hayes’ model 8. When the perceived certainty of the depicted environmental changes was entered as the mediator, the index of moderated mediation was significant (index = -1.16, SE = 0.52, 95% CI [-2.25, -0.20]; see also S1 Table and Fig 2 below). This suggests that the indirect effect through certainty was dependent on temporal framing condition. The indirect effect of political orientation was significant and negative across both conditions, but was stronger in the future condition (b = -2.62, SE = 0.44, 95% CI [-3.52, -1.82]) than the past condition (b = -1.45, E = .40, 95% CI [-2.28, -0.70]. This suggests that conservatives were relatively more certain that climate change is real in the past-focused condition *because* they were less skeptical about the human causes of the environmental changes they observed in the images than those in the future-focused condition.

Replicating this model with temporal distance of climate change in place as the mediator instead returns a non-significant index of moderated mediation (index = -0.92, SE = 0.86, 95% CI [-2.58, 0.80]), indicating that the effectiveness of the past-comparison frame for conservatives is not explained by differences in the perceived temporal distance of climate change.

Using model 15 to test for the potential mediation by nostalgia or past positive time perspective returned a non-significant index of moderated mediation for both nostalgia (index = -0.055, SE = 0.103, 95% CI [-0.309, 0.111]) and past-positive time perspective (index = -0.049, SE = 0.355, 95% CI [-0.797, 0.623]). These results suggest that the temporal framing effect by conservatism was not explained by individual differences in affinity for the past: either as indexed by past-positive feelings (i.e., nostalgia) or past-positive time perspective.

To further explore the possible role of the proposed explanatory variables, we conducted a series of exploratory analyses, reported in Table 2. In these, we entered two of the explanatory variables (perceived certainty, psychological distance) as dependent variables in the same regressions described above. It is only sensible to do this for the variables measured after the manipulation, as anything before this cannot be influenced by the frames. This is the first step Baldwin and Lammers [9] took when exploring potential explanations for their effects, and does allow a partial test of whether political orientation and framing condition affected the perceived certainty ratings and psychological (temporal) distance of climate change.

**Table 2:** Standardized regression coefficients regressing each explanatory variable on political orientation, condition, and the interaction term.

	Certainty ratings	Psychological (temporal) distance
**Step 1**	R^2^ = .110***	R^2^ = .213***
Political orientation	-.332***	.456***
Condition	-.011	.074
**Step 2**	ΔR^2^ = .009*	ΔR^2^ = .002
Political orientation	-.048	.321**
Condition	.181*	-.017
Political orientation X condition	-.353*	.168

The analyses of certainty ratings showed a significant interaction, which we followed up using the Johnson-Neyman technique and graph in S1 Fig, indicating a significant difference (*p* < .05) between certainty ratings of those exposed to the past- and future-focused conditions beyond a score of 5.9 on the liberal-conservative scale. This means that the difference in certainty ratings by condition only differed for conservatives at the more extreme end (*N* = 113). Individuals who identified as moderately conservative to very conservative were significantly more certain that environmental changes *have* occurred (i.e., if they were in the past-focused condition), than they *will* occur (future-focused condition).

These results provide further support for the certainty explanation, and again refute the psychological distance explanation. Political orientation was a significant predictor of perceptions of psychological distance, indicating that greater conservatism is related to a greater perception that climate change effects will occur further into the future, if at all. However, political orientation and condition did not interact to inform perceptions of psychological distance, and therefore this is an unlikely explanation for the temporal framing effect.

Lammers and Baldwin [42] suggested that nostalgia (indexed by the experience of positive past-focused feelings) explains the temporal framing effect, as conservatives experience greater nostalgia and subsequently are more affected by past comparisons. We wanted to expand on the analysis of the potential temporal attitudes-conservatism association by including the dimensions of time perspective as potential explanations for the temporal framing effect, and indeed could only test this explanation for the certainty of climate change outcome variable, finding no evidence of mediated moderation. In the absence of the expected political orientation * condition interactions, we conducted further exploratory analyses on the relationship between political orientation and the dimensions of time perspective, nostalgia (as a past-directed feeling), and future-directed feelings across the entire sample. For these analyses, we collapsed across framing condition because the measures were taken prior to the experimental manipulation.

The correlations in Table 3 show that conservative individuals endorsed greater positive time perspective (both past-positive and future-positive), and lower negative time perspective (both past-negative and future-negative), while political orientation was unrelated to present time perspective. Interestingly, we did not find any evidence of a relationship between conservatism and nostalgia. This is surprising, given the larger sample size in our study, and that we used the same measure of nostalgia as Lammers and Baldwin (Study 5 [42]). However, we measured nostalgia before the manipulation, and it is possible that this kind of nostalgia (state nostalgia) is only heightened in conservatives after exposure to past comparisons. The correlation analyses provide some evidence that conservatives may think to both the past and future more often more positively than their liberal counterparts.

**Table 3:** Associations between political orientation and the dimensions of time perspective.

	1.	2.	3.	4.	5.	6.	7.	8.
1. Political orientation								
2. Past positive time perspective	.27 [.19, .35]							
3. Past negative time perspective	-.20 [-.28, -.12]	-.12 [-.21,.-.04]						
4. Present time perspective	-.05 [-.14, .04]	.19 [.10, .27]	.05 [-.05, .15]					
5. Future positive time perspective	.21 [.13, .30]	.17 [.09, .26]	-.13 [-.21, -.05]	-.12 [-.21, -.03]				
6. Future negative time perspective	-.24 [-.32, -.16]	-.10 [-.18, -.02]	.42 [.35, .49]	.14 [.06, .22]	-.36 [-.43, -.28]			
7. Past-focused feelings	-.03 [-.12, .06]	.37 [.30, .45]	.33 [.25, .40]	.17 [.09, .26]	-.03 [-.12, .06]	.21 [.13, .29]		
8. Future-focused feelings	.09 [.00, .18]	.24 [.16, .32]	-.28 [-.36, -.20]	.27 [.18, .35]	.29 [.21, .37]	-.46 [-.52, -.39]	.06 [-.03, .16]	

*Note*. Political orientation was coded such that higher scores represent greater conservatism. Square brackets contain 95% confidence intervals based on 5000 bootstrap iterations.

### Dimensions of social dominance

In the next set of exploratory analyses, we tested whether the temporal framing effect interacted with each of the two dimensions of SDO. Anti-egalitarian (SDO-E) attitudes, which are usually more strongly tied to environmental responses, did interact with temporal framing condition for climate change certainty ratings and mitigation and adaptation policy support (Table 4). However, there was no interaction with SDO-D to predict these or other outcomes (see S2 Table).

**Table 4:** Standardized regression coefficients regressing each DV on SDO-E, condition, and the interaction term.

	Climate change belief	Climate change certainty	Climate change causes	Willing to sacrifice	Support mitigation	Support adaptation
**Step 1**	R^2^ = .312***	R^2^ = .256***	R^2^ = .228***	R^2^ = .204***	R^2^ = .285***	R^2^ = .208***
SDO-E	-.559***	-.500***	.478***	-.449***	-.533***	-.453***
Condition	-.069	-.105**	.013	-.081*	-.073*	-.083*
**Step 2**	ΔR^2^ = .004	ΔR^2^ = .007*	ΔR^2^ = .000	ΔR^2^ = .005	ΔR^2^ = .009*	ΔR^2^ = .008*
SDO-E	-.378**	-.249*	.432***	-.242*	-.250*	-.191
Condition	.054	.067	-.019	.060	.121	.096
SDO-E X condition	-.221	-.309*	.056	-.254	-.348*	-.321*

**Fig 3:**
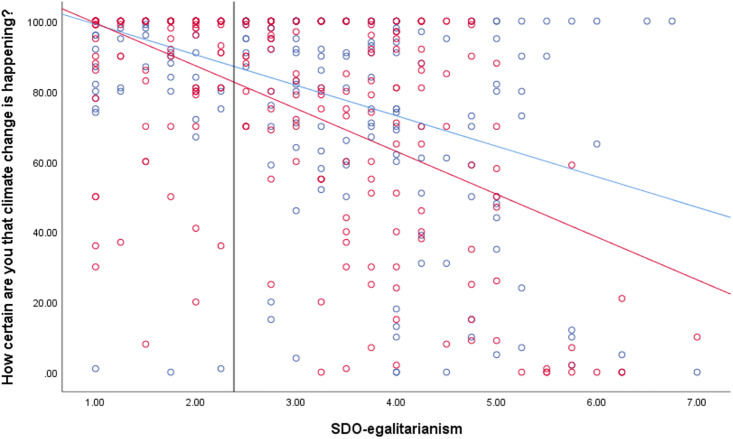
Association between anti-egalitarianism and certainty that climate change is happening by temporal framing condition (past = blue, future = red). Solid vertical line represents the Johnson-Neyman value. To the right of this, the differences in certainty ratings by condition are significant.

We followed up the significant SDO-E*condition interactions using Johnson-Neyman analyses in R. These showed that the difference by conditions became significant at the *p* < .05 level beyond a score of 2.38 (*N* = 510) on the SDO-E scale for climate change certainty, as graphed in Fig 3 below. Differences were significant beyond a score of 2.83 (*N* = 504) for both mitigation and adaptation support, which are graphed in S2 and S3 Figs. These differences indicate that for those who are relatively more tolerant of inequality (i.e., score higher in SDO-E), perceived certainty of climate change and support for both types of action on the issue increase after viewing the past-focused image pairs than the future-focused image pairs. Meanwhile, those who strongly rejected inequality (i.e., scored lower in SDO-E) had uniformly high support for action and certainty that climate change is happening, regardless of framing condition.

We then followed up these significant interactions using moderated mediation to test our explanations, again using model 8 to test the certainty and temporal distance explanations, and model 15 to test the nostalgia and past-positive time perspective explanations. Again, assumptions of linearity, homoscedasticity and normality were tested for all models and met. Tolerance and VIF were all within acceptable cut-offs (<1.05 in all cases). We did not find evidence that any of the explanatory variables explained the relatively greater certainty that climate change is happening among those higher in SDO-E in the past-focused condition (certainty of environmental changes: index = -1.04, SE = 0.77, 95% CI [-2.61, 0.44]; temporal distance: index = -1.34, SE = 1.30, 95% CI [-3.81, 1.24]; nostalgia: index = -0.03, SE = 0.12, 95% CI [-0.33, 0.16]; past-positive time perspective: index = -0.14, SE = 0.25, 95% CI [-0.70, 0.30]). We again did not find support for any proposed mediators when these models were repeated for mitigation policy support (certainty: index = -0.03, SE = 0.02, 95% CI [-0.07, 0.01]; temporal distance: index = -0.04, SE = 0.04, 95% CI [-0.12, 0.04]; nostalgia: index = -.00, SE = .004, 95% CI [-.01, .01]; past-positive time perspective: index = .00, SE = .01, 95% CI [-0.02, 0.02]) or adaptation policy support (certainty: index = -0.02, SE = 0.02, 95% CI [-.05, .01]; temporal distance: index = -0.03, SE = 0.03, 95% CI [-0.10, 0.03]; nostalgia: index = .00, SE = .00, 95% CI [-0.01, 0.01]; past-positive time perspective: index = 0.00, SE = 0.01, 95% CI [-.02, 0.02]).

As multinomial logistic regression runs the analysis at each level of the IV, which is inappropriate for SDO mean scores (i.e., this would involve 25 levels of SDO-E), we instead used binary logistic regression to test the likelihood of signing the pro-climate petition (*N* = 219) or neither petition (*N* = 301), with those who agreed to sign the hoax petition (*N* = 15) removed from this analysis. As the dependent variable was recoded to be dichotomous, the independent variables were continuous (SDO-E), binary (condition), and unrelated to each other, and we passed the Box-Tidwell test (interaction between SDO-E and its log was not a significant predictor, *p* = .068), we met assumptions of binary logistic regression. The model with SDO-E and temporal framing condition as predictors was significant (χ^2^(2) = 57.25, *p* < .001) and explained 14% of the variance in agreement to sign a petition (Nagelkerke R^2^ = .140), and correctly classified 62.7% of cases. Each increase in SDO-E scores was associated with a decrease in the likelihood of signing the pro-climate petition (Odds ratio = .588; *p* < .001), but temporal framing condition was unrelated (*p* = .150). Adding the interaction term explained less than one percent of additional variance, and this was not significant (χ^2^(1) = 1.98, *p* = .160). Results were similar for SDO-D (Step 1: χ^2^(2) = 38.28, *p* < .001, Nagelkerke R^2^ = .095, SDO-D Odds ratio = 0.633 Step 2: χ^2^(1) = .08, *p* = .774).

### Summary and conclusions

To summarize, throughout our analyses we found a reliable effect of ideology on environmental outcomes, which held regardless of whether ideology was operationalized as political orientation, anti-egalitarianism (SDO-E), or dominance (SDO-D). The effect of temporal framing condition itself was unreliable (either weak or non-significant), and rarely interacted with ideology. Where there was a significant interaction, the only explanation for which we found support was the certainty explanation: when the past-focused frame increased conservative’s pro-environmentalism, it did so by increasing confidence that the environmental changes depicted in the images had occurred. Despite detecting the interaction more reliably when including SDO-E in place of political orientation, results from a series of moderated mediations did not support any of the explanatory variables.

There are several possibilities for the failure to detect interaction effects in this study. First, we may not have found evidence for the temporal framing effect because the frame itself, and therefore the manipulation we employed, is not effective in motivating conservatives’ environmentalism. If this explanation is correct, it highlights the importance of replication for interrogating how robust our effects are, especially when the goal is to inform climate change communications. If the effects are not strong enough to be detected immediately after presenting the manipulation, we have little confidence they would increase acceptance of, and sustain action on, climate change if used in widespread public advertisement campaigns.

As Baldwin and Lammers [9] did present a series of conceptual replications of their temporal framing effect, the alternative possibility is that the effect exists, but did not emerge in our study due to differences in the study design. For instance, the context under which participants rated the photo pairs differed in our study. Baldwin and Lammers asked participants to rate each image pair on the extent they demonstrated the effects of climate change. We instead asked participants to reflect on the most likely causes (human versus natural) of each observed environmental change, which may have induced greater scepticism. This would be consistent with the processing style moderation included in Lammers and Baldwin’s (Study 7 [42]) research. They suggested that the effects of temporal focus should be stronger when participants evaluate the message on superficial features (such as liking) compared to when the content is carefully evaluated. To test this, they presented participants with a message aimed to increase leniency in criminal justice, and asked their endorsement of the message. However, before rating their endorsement, some participants were asked to focus on understanding the message (to engage central processing) while others were not (peripheral processing). Those who were encouraged to interrogate the meaning of the message were not swayed by the temporal framing effect.

Another explanation for the non-replication is the differences in our outcome measures. Baldwin and Lammers [9] used the NEP scale, which is the most widely used measure of pro-environmentalism [49]. However, there are criticisms of the scale, including whether it ought to be treated as measuring attitudes, beliefs, values, or a particular worldview, leading to calls to develop a new standard measure [50]. If the temporal framing effect increases endorsement only of general pro-environmentalism, this suggests that the effect cannot be used to motivate intentions to sacrifice or to increase policy support, both of which are important in addressing climate change. The fact that our results were not consistent across the multiple indicators of environmentalism we had in our study adds weight to this, though we did not include the New Environmental Paradigm scale to see if the effect emerges on this outcome measure.

Lastly, the political context in the United States at the time of our study was different from that of Baldwin and Lammers [9]. When Baldwin and Lammers asked participants to think to the past, Obama was president; our data were collected under the Trump administration, which campaigned on a return to the past (e.g. “Make America Great Again”). It is possible that past comparisons are no longer effective for conservatives in this context, as they have already returned to their ‘desired’ past. This last possibility is not readily testable, so instead we sought to avoid this potential limitation of our first study by recruiting participants from the United Kingdom. Importantly, the theoretical arguments are not localised to one country or another, but ought to apply anywhere. Supporting this, Lammers and Baldwin’s [42] research that broadens the temporal framing effect to other social issues drew from samples in the United States, United Kingdom, and Germany.

## Study two

Study Two builds on our findings from Study One and tests the potential explanations for the failure to replicate the temporal framing effect. We again included the manipulation from Baldwin and Lammers (Study 3 [9]), randomly allocating participants to the same image-based temporal focus manipulation (past- versus future-comparisons). Participants were also randomly allocated to rate the image comparisons based on their likely causes (human versus natural, as in Study One), or the extent that each image pair depicted the effects of climate change (as in the original research).

Our hypotheses were preregistered on the Open Science Framework (https://osf.io/v97jc), with the first two based on Baldwin and Lammer’s [9] research. We expected a main effect of political orientation such that political conservatism predicts lower environmentalism (across all outcome variables; H1). Next, where there is a significant interaction effect of temporal framing by political orientation, we expected that the past focused environmental images would attenuate the negative effect of political orientation on environmentalism compared to future focused images (H2).

Our next set of predictions were conditional and based on the potential explanations for our failure to replicate the temporal framing by political orientation interaction. If asking participants to rate the likely causes of environmental changes inoculated them against the temporal framing interaction, then we expect to replicate Baldwin and Lammer’s [9] temporal framing by political orientation interaction among those participants who rated the extent the image pairs depicted climate change, and not among those rating the likely causes of the environmental changes depicted (H3).

We include the same host of outcome variables from Study One, as well as the NEP, for consistency with Baldwin and Lammers [9]. If the temporal frame only moderates the effect of political orientation on pro-environmental attitudes, we expected to replicate the interaction only when scores on the NEP are included as the dependent variable, and not for the other outcome variables (H4).

The main purpose of Study One was to test different explanations for the temporal framing effect interaction, which is only possible if this interaction is detected in Study Two. If so, we expect that the interaction between temporal framing condition and political orientation will be explained (mediated) by participants’ ratings of how certain they are that the changes had happened (past-focused condition) or will happen (future-focused condition). Specifically, we predicted that participants who are more politically conservative would report increased environmentalism after viewing the past comparisons (versus future comparisons), and this effect would be explained by greater ratings of certainty (H5).

Finally, we build on the promising findings highlighting the importance of SDO-E by also incorporating the dimensions of RWA. Lammers and Baldwin [42] referred to conservatives’ “preference to maintain the past” (p. 600), which is a similar concept to the traditionalism component of RWA. Agreement with items such as “The "old-fashioned ways" and "old-fashioned values" still show the best way to live” form a greater preference of traditionalism. Lammers and Baldwin (Study 5 [42]) also suggested that nostalgia explains conservatives’ preference for ‘old fashioned values’, which are directly measured using an RWA-Traditionalism measure. Due to the stated limitations of measuring political ideology by asking participants to identify themselves on a single continuum from liberal to conservative, utilising the RWA-Traditionalism measure enables us to directly capture this important component of conservative ideology. Analyses involving these dimensions of ideology are exploratory; however, Study One suggests SDO-E is the key dimension of social dominance, and the stronger grounding in a preference for things of the past indexed by RWA-Traditionalism suggests this will be the more relevant dimension of authoritarianism in this context.

## Method

### Participants

Following the same approach as Study 1, participants were again recruited via Prolific and compensated US$1.83 for completing the ten-minute survey. As Study Two included a second independent variable (with 2 levels), we aimed to double our desired sample size to maintain at least 250 participants per condition. We aimed to recruit at least 1000 participants and posted 1100 places to account for possible non-response (i.e., a 10% buffer), and 1102 individuals completed the survey. Participants were 34.7 years old on average (SD = 13.26, range 18–78), with a roughly even gender distribution (54% female, 45.5% male, 0.5% other). Once again to ensure we recruited a roughly even distribution of individuals across the political spectrum, we alternated between open recruitment and recruiting conservatives only. Consistent with Study One, varying the posting of places obtained an approximately normal distribution of political ideology (range = 0/left-wing to 10/right-wing; M = 4.87, SD = 2.32).

### Materials and procedure

#### Part I

Participants completed the same two-dimensional measure of SDO [61] as in Study One (SDO-D α = .74; SDO-E α = .80), a single item political orientation scale (ranging from 0 to 10), and the same measure of time-perspective [65] for the past-positive dimension only (α = .77). Participants also completed the ACT scale [74] to measure the three components of RWA (Authoritarianism α = .84; Conservatism α = .88; Traditionalism α = .79).

#### Part II

Participants were randomly allocated to either the past- or future-focused condition, and shown the same series of 14 photograph pairs used in Baldwin and Lammers [9] and Study One above. To test whether the way they were asked to rate the images affected responses, participants were randomly allocated to rate either the extent to which they believed the changes depicted in each photograph set were caused by humans (as in Study One; Causes condition) or the extent to which each image demonstrated the effects of climate change (as in [9]; Effects condition).

#### Part III

Participants were then presented with Loy and Spence’s [75] three item temporal distance of climate change scale (α = .75), Baldwin, White and Sullivan’s [70] three items measuring nostalgia (α = .83) and three items used in Study One to measure future focused emotions (α = .92).

Next, the environmental variables were presented in a randomised order. Consistent with Study One, we measured climate change belief (2 items, *r* = .65), willingness to sacrifice (2 items, *r* = .83), mitigation support (α = .85), adaptation support (α = .83), and willingness to sign a petition. We also included the 15-item revised NEP scale [76], consisting of 8 items measuring endorsement of the new environmental paradigm (α = .79) and 7 the dominant social paradigm (α = .77).

Finally, participants were asked to reflect on how certain they are that the images depicted in the photographs have happened (past-focused condition) or will happen (future-focused condition). Responses are recorded on a sliding scale from 0 (not at all certain) to 100 (completely certain). This measure was moved to the end of the survey to ensure it could not influence responses to the environmental outcome variables.

### Results

As with Study 1, we tested assumptions of regression analysis for all moderated multiple regressions conducted for the main analysis. Once again, there were no major violations of normality or homoscedasticity. Again, as there was only one continuous independent variable in the models, multicollinearity was not a concern.

S3 (for participants in the effects rating condition) and S4 Tables (causes rating condition) in the supplementary materials report results where each environmental outcome variable is regressed onto condition (past, future), political orientation, and the interaction term (condition * political orientation). Supporting our first hypothesis, these results show that across both rating conditions, political orientation was a consistent negative predictor of pro-environmentalism, exhibiting small to moderate effects on pro-environmental attitudes, climate change belief and certainty ratings, willingness to make sacrifices, and support for mitigation—but not adaptation—policy.

There were no significant interactions between political orientation and temporal framing, regardless of rating condition. Moderated moderation analysis using Model 3 of the PROCESS macro [73] confirmed that, with political orientation as the predictor variable, rating condition (effects versus causes) did not significantly interact with temporal frame (past versus future) on any environmental outcome variable (Pro-environmental attitudes: *p* = .700; Climate change belief: *p* = .144; Climate change certainty: *p* = .551; Climate change causes: *p* = .051; Willingness to sacrifice: *p* = .629; Mitigation policy support: *p* = .693; Adaptation policy support: *p* = .281). We therefore did not find evidence that the way participants were asked to evaluate the image pairs influenced their responses. This justified collapsing across rating conditions to test the effect of the temporal frame only, thereby increasing sample size to offer a higher-powered test of the potential temporal framing effect interaction.

**Fig 4:**
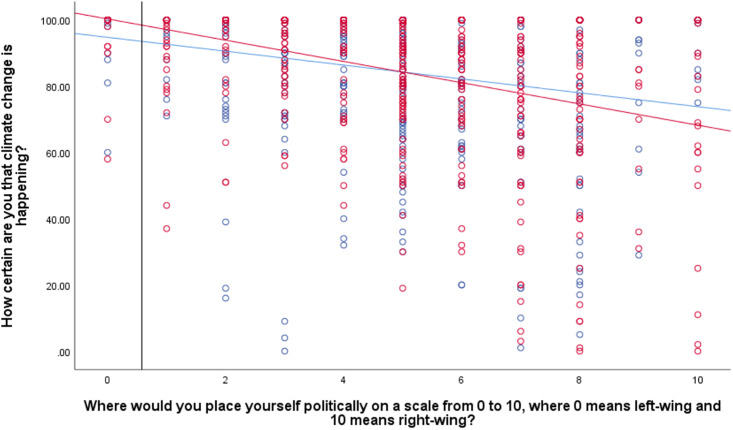
Association between political orientation and certainty that climate change is happening by temporal framing condition (past = blue, future = red). Solid vertical line represents the Johnson-Neyman value. To the left of this, the differences in certainty ratings by condition are significant.

These results are presented in Table 5 below, and show a significant temporal frame by political orientation interaction only on the ratings of climate change certainty. Fig 4 guides interpretation of this significant interaction. This shows that conservatives in the past-focused condition appeared to report slightly higher certainty that climate change is happening than conservatives in the future-focused condition. However, Johnson-Neyman analyses show a significant difference in certainty ratings by temporal focus condition only for *left-wing* individuals who score below 0.58 (i.e., place themselves a 0 on the scale, N = 39), and no significant difference at the right-wing side of the spectrum.

**Table 5:** Standardized regression coefficients regressing each DV on political orientation, condition, and the interaction term all participants, independent of rating condition.

	Pro-environmental attitudes	Climate change belief	Climate change certainty	Climate change causes	Willingness to sacrifice	Support for mitigation policy	Support for adaptation policy
**Step 1**	R^2^ = .023***	R^2^ = .128***	R^2^ = .091***	R^2^ = .088***	R^2^ = .095***	R^2^ = .067***	R^2^ = .002
Political orientation	-.153***	-.357***	-.301***	.294***	-.309***	-.257***	-.039
Condition	-.004	.045	.003	-.050	.007	.034	.023
**Step 2**	ΔR^2^ = .001	ΔR^2^ = .001	ΔR^2^ = .004*	ΔR^2^ = .000	ΔR^2^ = .001	ΔR^2^ = .001	ΔR^2^ = .000
Political orientation	-.047	-.270**	-.110	.292**	-.202*	-.154	-.040
Condition	.068	.105	.134*	-.052	.080	.105	.022
Political orientation X condition	-.135	-.110	-.244*	.003	-.136	-.132	.002

Note.

*** p < .001,

** p < .01,

* p < .05

Finally, we compared the likelihood of agreeing to sign the pro-climate (*N* = 380), hoax (*N* = 44), or neither petition (*N* = 678) using multinomial logistic regression following the same model as in Study One. Model fit was satisfactory, with a non-significant Pearson Chi-Square value (χ^2^ (36) = 43.44, *p* = .184) and the model explaining more variance in the dependent variable than an intercept-only model (χ^2^(6) = 47.16, *p* < .001). However, this may be due to the large sample size, as only 5% of the variance was explained (Nagelkerke pseudo R^2^ = .053). Both the interaction term (*p* = .497) and temporal framing condition (*p* = .570) variables were non-significant predictors and subsequently removed from the analysis to improve model fit (Pearson Chi-Square (18) = 21.34, *p* = .263; Model fit: (χ^2^(2) = 45.29, *p* < .001). Consistent with the first study, relative to those who did not want to sign either petition (reference category), those who agreed to sign the petition acknowledging the reality of climate change were less conservative (B = -.171, *p* < .001) and those agreeing to sign the hoax petition were more conservative (B = .144, *p* = .039). For each 1-unit increase in political conservatism, participants were less likely to sign the pro-climate petition (Odds ratio = 0.84) and more likely to sign the hoax petition (Odds ratio = 1.16).

We had planned to test whether nostalgia, past-positive time perspective, temporal distance of climate change, and ratings of certainty the environmental changes had happened/will happen as mediators of the temporal framing effect (i.e., using moderated mediation). These analyses could only be conducted following a temporal framing interaction effect. Although the interaction was detected for climate change certainty, the significant interaction occurred on the *liberal* end. As the proposed mediators were planned as explanations of a conservative bias, follow up analyses were not conducted as planned. Instead, we ran a series of (non-preregistered) exploratory hierarchical regressions as in Study One. The possible explanatory variables were entered as outcome variables to test whether responses depended on the interplay between political orientation and temporal framing condition. These analyses, reported in S5 Table, show that political conservatism predicted a decrease in certainty about the environmental changes, lower agreement that climate change effects will occur in the future (temporal distance), and higher past positive time perspective. Participants in the past condition reported slightly higher nostalgia than those in the future condition; however, political orientation was *unrelated* to nostalgia, and there were no significant interactions between political orientation and framing condition.

### Exploratory analyses on the dimensions of conservative ideology

To explore the possible roles of the dimensions of SDO and RWA in the temporal framing effect, exploratory analyses were conducted where these were entered in place of political ideology as predictors of each continuous outcome variable and are presented in the Supplementary Materials, and described here.

**Fig 5:**
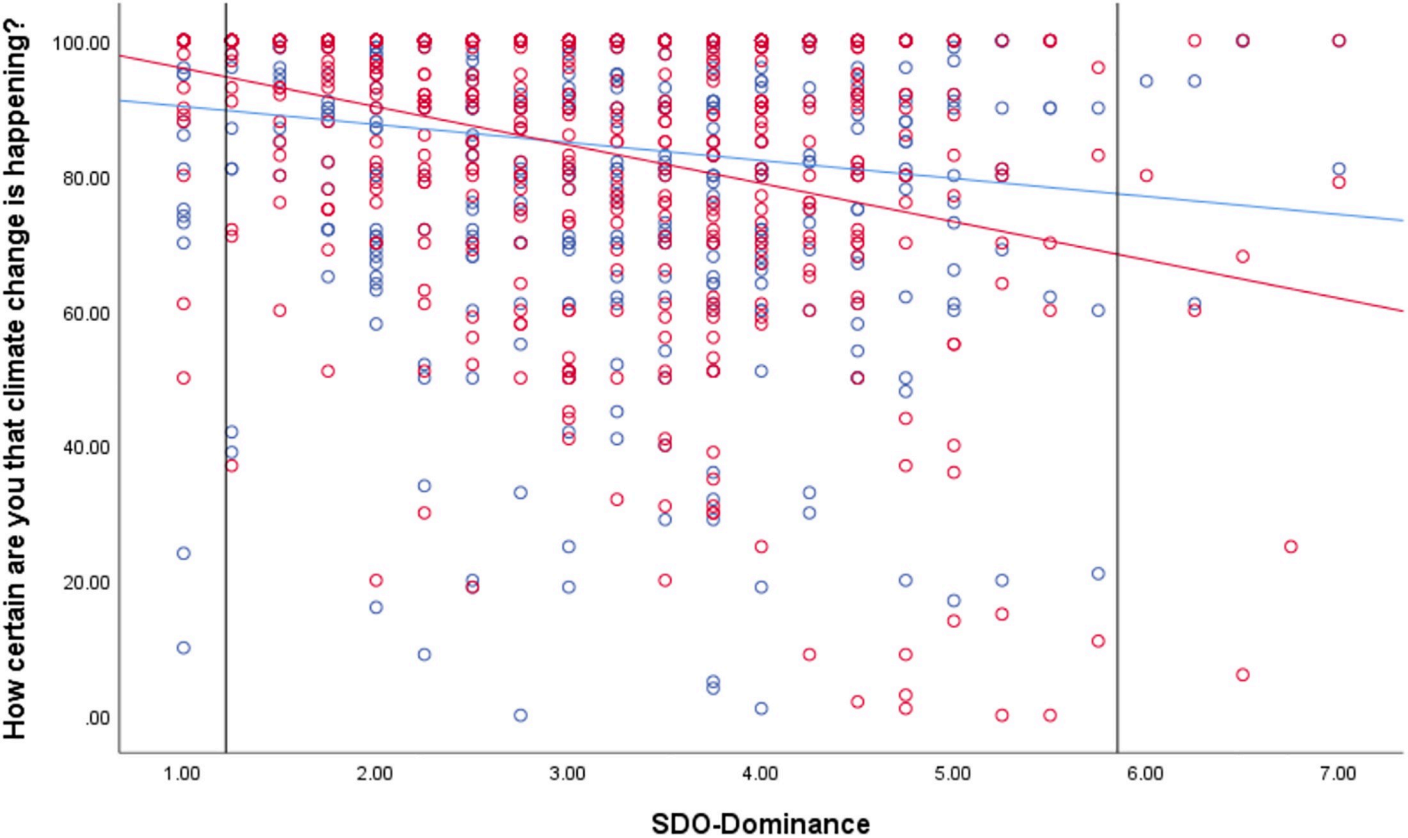
Association between SDO-D and certainty that climate change is happening by temporal framing condition (past = blue, future = red). Solid vertical lines represents the Johnson-Neyman value. Outside of these bounds, the differences in certainty ratings by condition are significant.

Both dimensions of social dominance predicted lower environmentalism. While SDO-E did not interact with the temporal frame to predict any environmental outcome variable (S6 Table), SDO-D interacted with temporal frame on climate change certainty ratings and willingness to make sacrifices for the environment (S7 Table). Johnson-Neyman analyses returned significant differences between temporal framing condition for individuals scoring below 1.22 (i.e., participants who strongly disagree to all items, *N* = 68) or above 5.85 (overall agreement to strong agreement, *N* = 16) with SDO-D. This means that those who endorsed group dominance were more certain that climate change is happening after viewing the series of images comparing the current state of the environment to the past compared to those who viewed images comparing current and future state of the environment (though note this only applied to a small subset of the overall sample, and appears in Fig 5 to be driven by just a few very uncertain highly dominant participants). Conversely, for individuals who strongly *oppose* intergroup dominance, perceived certainty of climate change is higher for those who viewed future-focused comparisons.

As our preregistered analyses of our potential explanations for the temporal framing interaction were not possible due to the lack of interaction with political orientation, we conducted these analyses with SDO-D to follow up the significant interaction reported here. However, we did not find support for any of these explanations (Model 8: Certainty of environmental changes: index = -.48, SE = .50, 95% CI [-1.51, .49]; Temporal distance: index = -.38, SE = .28, 95% CI [-.96, .12]; nostalgia: index = .05, SE = .07, 95% CI [-.07, .20]; Model 15: Past positive time perspective: index = .18, SE = .14, 95% CI [-.06, .48]).

For those largely indifferent to, or supportive of intergroup dominance (scoring above 3.87 on SDO-D, *N* = 287), there was a significant difference between conditions on willingness to make sacrifices for the environment, with those observing the past changes more willing to sacrifice than those viewing future changes (graphed in S4 Fig). Again, the differences in responses to past- and future-focused frames could not be explained by certainty of environmental changes (index = -.03, SE = .03, 95% CI [-.08, .03]), perceived temporal distance of climate change: index = -.02, SE = .02, 95% CI [-.05, .01]), nostalgia (index = .01, SE = .01, 95% CI [-.01, .03]) or past positive time perspective (index = .00, SE = .00, 95% CI [-.00, .00]).

Authoritarian aggression (S8 Table), conservatism (S9 Table) and traditionalism (S10 Table) each related to lower environmentalism, but no component of RWA interacted with temporal frame to do so.

Finally, given the minimal effects of temporal framing condition and the interaction terms on environmentalism overall, our last set of exploratory analyses compared the relative strength of the dimensions of SDO and RWA in predicting each environmental variable, irrespective of experimental condition (S11 Table). The pattern of unique associations was consistent with those reported elsewhere [62], and showed SDO-E and Traditionalism consistently predicted lower pro-environmentalism, while the other dimensions were weak and inconsistent predictors.

## Summary and conclusions

In Study Two, we substantially increased the sample size and therefore power to detect even weak effects, closely adhered to Baldwin and Lammers’ [9] study design, and took steps to address potential reasons for the failure to replicate the temporal framing effect in Study One. Our results still do not support the image-based temporal framing manipulation as an effective frame to induce pro-environmentalism among conservatives.

The effects we did detect were weak, and although the overall sample size was much larger than typical framing studies and those employed by the temporal framing literature, the significant differences were only driven by a few participants at the ideological extremes. The pattern of results was reasonably consistent with Study One, showing that the most reliable interaction between ideology and temporal frame was on climate change certainty. However, in this study, the interaction was significant for liberal individuals (not conservative) when ideology was indexed by self-placement on a liberal-conservative continuum. This indicates that a past-focused frame may backfire among political liberals, who are relatively more certain of the reality of climate change after viewing images depicting how the environment will change in future than after viewing images depicting how the environment has changed from the past. Given this is the first study to demonstrate such an effect on environmental attitudes, it is important future work also aims to replicate this backfire effect in liberal individuals.

Findings from our exploratory analyses on the dimensions of social dominance and authoritarianism contribute to the growing literature on their associations with environmentalism [62, 63]. Consistent with research on social dominance, we demonstrate that attitudes in favour of the unequal distribution of resources across groups (SDO-E), or the oppression of low-status groups by high-status groups (SDO-D), are associated with lower pro-environmentalism. Participants invested in tradition, following those in power, and supporting the persecution of deviants (i.e., the dimensions of right-wing authoritarianism) also tended to report lower pro-environmentalism. When pitted against each other, we replicate recent findings that of these dimensions, SDO-E and Traditionalism demonstrate strongest unique associations with environmentalism [62].

While no aspect of RWA interacted with temporal framing condition, we unexpectedly found that SDO-D did. Those strongly pro- or anti-intergroup dominance responded differently to the manipulation. Participants who opposed intergroup dominance experienced a decrease in climate change certainty after viewing past-comparisons (relative to those who viewed future-comparisons), while those supportive of intergroup dominance experienced an increase in certainty. This finding partially aligns with the evidence for a backfire effect among those holding more liberal values, and suggests this may occur due to individual differences in support for intergroup dominance.

The most promising evidence for the efficacy of the temporal comparison manipulation was the increase in willingness to make sacrifices for the environment among participants indifferent to, or supportive of, intergroup dominance who viewed the past-focused images. This effect was also driven by a larger sample than the effects on certainty (*N* = 287 versus Ns of 39 and 68). However, this effect was not detected in Study One, where the same variables were included, and therefore may not be reliable. Further research ought to explore this effect further, and consider possible reasons why those who support intergroup oppression may respond more favourably to past focused images, as none of our exploratory analyses testing potential mediators explained this effect.

We did not find support for any of the possible explanations for the failure to replicate the temporal framing interaction in Study One. Specifically, the different rating conditions did not moderate the effect, suggesting that asking participants to consider the possible causes of environmental changes or the extent these changes depict climate change either does not differentially engage central and peripheral processing or that this temporal framing effect does not depend on processing style. The manipulation also did not increase pro-environmentalism as indexed by the New Environmental Paradigm scale. Therefore, it does not appear to be the case that the temporal framing effect acts only on this broad measure of pro-environmentalism, and was thus not detected on the range of outcome measures included in Study One. Instead, where the temporal frame did interact with ideology, it was detected on the ratings of climate change certainty (political orientation and SDO-D) or willingness to make sacrifices for the environment (SDO-D). While this offers limited support for our suggestion that a change in certainty explains why the temporal framing effect may be effective for conservatives, we found little evidence of the temporal framing effect itself, and therefore we were not able to test the other proposed explanations of the effect.

## General discussion

Our research aimed to replicate the temporal framing effect, and extend this literature by evaluating the potential psychological mechanisms that may underlie conservatives’ divergent responses to past- and future-focused frames. However, we found limited evidence of a temporal framing effect, with no shift in support for climate change policy or pro-environmental attitudes in response to the frames. The temporal framing effect appeared to motivate perceived certainty that climate change is real, with conservatives in Study One more certain after viewing environmental changes that happened (past condition) than changes that will happen (future condition). However, while previous research suggests liberals are as receptive to past- and future-focused frames, and therefore the temporal manipulation ought to only unite pro-environmentalism, we instead found a backfire effect for liberals in Study Two: liberals presented with a past-focused frame were *less certain* that climate change was real.

Taken together, these findings cast some doubt on the utility of the temporal framing effect in reducing the ideological gap in important aspects of pro-environmentalism. Our research implicates a past-focused frame in potentially undermining liberal pro-environmentalism, and having only weak effects on conservative environmentalism that are inconsistent across indicators of environmentalism, dimensions of ideology, and across studies.

### Reproducibility of message framing research

In message framing studies, researchers emphasize different aspects of an issue in attempts to persuade the reader [77]. Framing studies in environmental psychology test different ways of communicating about environmental issues such as climate change, to be used as a basis of broader message campaigns or interventions. These message frames require careful development, and testing on different segments of the population, as they are sometimes ineffective or backfire to reduce environmentalism for some groups [21, 28, 30, 36, 78]. While innovative message frames ought to be encouraged to expand our repertoire of environmental communication strategies, we argue that it is equally important to refine this repertoire through large-scale replication.

Despite the importance of replication to the scientific process, peer-reviewed published replications of existing environmental framing studies are rare, reflecting a wider problem in the psychology discipline: just over one percent of articles published in the top 100 psychology journals are replications [79]. A recent review of psychology journals found that only 3% explicitly stated that replications were accepted [80]. Replications are less publishable, less likely to attract grant funding, and less likely to lead to academic promotion, and therefore there is little incentive to conduct these studies [81, 82]. Research is more likely to be published if it shows evidence for an effect, rather than against it, and psychology has the highest proportion of positive results of the sciences (over 90% [83]). Publication bias has the potential to inflate the rate of published false positive findings, while simultaneously discouraging attempts to replicate existing research, making it difficult to quantify the extent of the problem. In one attempt to do this, the Open Science Collaboration [84] estimated that just 25% of social psychology findings replicate. Furthermore, this low replicability of psychological findings appears to diminish public trust [85, 86].

Related to this suite of issues surrounding publication bias are problems with sample sizes and statistical power within environmental framing studies. Many studies appear to have small sample sizes, which reduces the likelihood of detecting true effects (i.e., may return more false negatives), and can *increase* the risk of false positives [87]. This issue is exacerbated when examining interaction effects, which require substantially larger samples to be adequately powered, especially if an attenuation effect is hypothesised [88, 89]. We echo recent calls (e.g. [90]) for increased transparency in psychological research: preregistration of planned sample size, study design and analytic plan, and complete reporting of results. We are not aware of any cohesive and comprehensive attempt to replicate existing environmental framing studies. Because their findings have potential for informing broader climate change communications, we argue this is an important next step for environmental psychology. This line of research may require a change to research culture, including grant funding and publication priorities.

### Explanations for the (null) effects

As noted above, we largely failed to replicate a temporal framing effect across two studies. To be clear, this does not necessarily mean that the effect does not exist. Instead, our findings contribute to the range of evidence about the effect, and may usefully be incorporated into the meta-analysis reported in Baldwin and Lammers [9] to help refine the confidence interval around the overall effect size estimate for the temporal framing effect on conservatives’ pro-environmentalism.

We also note that Baldwin and Lammers [9] demonstrated evidence for the temporal framing effect in a series of conceptual replications. They manipulated temporal focus in diverse ways, such as by using written messages ostensibly written by previous participants (past: “Looking back to our nation’s past”; future: “Looking forward to our nation’s future”), or real environmental charities (past: “Restoring the planet to its original state”; future: “Creating a new earth for the future”). Successful replications are more common when the same authors are involved in the subsequent work (91.7% replicate) than when they are conducted by independent researchers (64.6% [79]), which could explain the discrepancy in the reproducibility of the finding. Alternatively, the visual comparison method we used might be less effective in inducing temporal comparisons than the other methods Baldwin and Lammers used. This could be tested in future replication projects by attempting to replicate the other temporal comparison manipulations.

At the time of writing, another independent group of researchers have had a manuscript accepted that reports on a replication of Baldwin and Lammers’ first study. Kim and colleagues [91] twice attempted to replicate the temporal framing interaction reported in Study 1 of [9], which asks participants to read a paragraph about acting on the environment that either appeals for action by comparing the present to the past (i.e., “we need to undo what we’ve done so that the world can go back to how it was…”) or comparing the present to the future (“we need to stop what we are doing so that the world can be what it’s supposed to be in the future”). Kim et al. conducted direct replications, preregistering their analyses and more than doubling the original study sample size (Ns of 538 & 541). However, they also failed to replicate Baldwin and Lammers’ results, finding no difference in conservative environmental attitudes in the past- and future-focused conditions. Together, the series of replications reported in our paper and in the Kim et al. paper casts greater doubt on the efficacy of temporal framing for motivating environmentalism, suggesting Baldwin and Lammers’ temporal framing effect is fragile or is highly temporally and geopolitically specific.

Another reason replication studies fail to detect effects is because they are often underpowered [92]. This is unlikely to be the case with our research, or Kim et al.’s [91], who reported statistical power above 98% for both studies. A sensitivity analysis based on our sample sizes suggest these were sufficiently powered to detect very small effect sizes (Study One: *f*^*2*^ of .015; Study Two: *f*^*2*^ of .007). Furthermore, our total sample was more than two (Study One) and five (Study Two) times larger than Baldwin and Lammers’ [9] original research, where significant interactions were detected. If the true effect of the particular temporal framing manipulation employed here is typically lower than this, then even if it can be detected by increasing the sample size, the practical significance of the effect is questionable. Furthermore, the recent Kim et al. study failed to replicate the effect with a similarly high-powered study.

We addressed some possible reasons for the nonreplication of the temporal framing effect in our second study, including the measures of environmentalism included, instructions for rating the temporal comparison images, and context of the study. The past comparisons still did not appear to motivate pro-environmental attitudes in Study Two, and we did not find evidence that asking participants to rate the likely cause of the environmental changes was responsible for neutralising the temporal framing effect. We were unable to directly test whether the context of the study (i.e. United States under a Trump presidency) explained why the temporal framing effect no longer interacted with political orientation. However, given that Study Two returned similar results among a sample recruited from a different context (the United Kingdom), there is little support for this explanation. This is consistent with findings from the Many Labs collaboration, which determined that the reproducibility of a finding is largely due to the effect examined, rather than the sample or context of the replication [93]. Future researchers may wish to explore whether other potentially moderating variables that were not included in our study may disarm the temporal framing effect.

While the temporal framing effect did not replicate in our studies, the associations between ideology and environmentalism were consistent with previous research and add to the literature on the ideological divide in pro-environmentalism that is observed in Anglosphere nations [1, 58]. Furthermore, the ineffectiveness of the temporal frame overall is consistent with psychological distance literature. Correlational research shows that individuals are more concerned about climate change when they believe its effects are psychologically *close*, including close in time (i.e., temporal distance [47]). However, attempts to manipulate psychological distance to draw the effects of climate change closer in time have shown inconsistent effects on pro-environmental attitudes and behaviours.

Previous studies showing no effect of temporal distance manipulations [48] generally do not consider whether these depend on political orientation. The exceptions to this do show interactions with political orientation, but they use different manipulations than those by Baldwin and Lammers [9], as they compare messaging about environmental risks happening now, or at varying degrees into the future. For example, conservatives in the United States are more supportive of climate policy when New York is portrayed as potentially unlivable *further* in the future (by 2066, rather than by 2020 or 2047 [94]), and show that positioning heightened risk from a zoological disease as temporally close (happening now) was more polarising than locating the risk further in the future [95]. Therefore, it is possible that conservatives are responding as well to future-focused messages as they are to past-focused messages, which is not noticeable in the absence of a present-focused frame. Given this, future research should also compare past- and future-focused frames to present-focus frames to obtain a fuller picture of these potential effects, and thoroughly review the overlap between temporal psychological distance research and Baldwin and Lammers’ [9] temporal comparison manipulations.

### Strengths and limitations

Our research contributes to the small but growing literature on the reproducibility of research in psychology, especially within the environmental psychology field. There were strengths in several aspects of the design of the research. This includes the large sample sizes used in both studies, which were conducted across two contexts where climate change is a politicized issue (Study One, the US; Study Two, the United Kingdom). We also included a range of environmental outcome variables, which enabled a test of the aspects of environmentalism potentially influenced by the manipulation (if any). Further still, we explored several reasons for the nonreplication in our second study, such as that the temporal framing effect may be sensitive to the context, way that participants engage with the frame by rating the images, and aspect of environmentalism measured.

As well as testing how robust the temporal framing effects are through replication, we explored why some frames effectively shift attitudes and behaviours of some groups. Lammers and Baldwin [27] suggested that nostalgia may underlie the efficacy of temporal comparison frames. However, we argued that conservatives may respond to a past-focused frame because changes that *have happened* are more certain than changes that *will happen*. The greater responsiveness to certainty-based manipulations would be consistent with those on the political right preferring certainty and cognitive closure over uncertainty [96]. If this explanation were correct, it could inform other climate change communications research outside of the temporal framing literature by focusing on manipulating certainty. We did find some support for the explanation, but the increased certainty about climate change after viewing past comparisons did not generalise to increase other types of environmental engagement such as belief in the human causes of climate change, pro-environmental attitudes, or policy support. This means either that increasing certainty does not usefully motivate other forms of environmental engagement, or that changing environmentalism requires a stronger increase in certainty.

We did not find support for the other potential explanations for the temporal framing effect, including nostalgia, past positive time perspective, and temporal distance. However, a limitation of our research is that a full test of these possible explanations was not possible, as the temporal framing effect itself was not reliably detected. We are also unable to offer a definitive explanation for *why* the temporal framing effect was not consistently found in our research. This leaves opportunities for further research on the replicability of other methods of inducing temporal comparisons to test the veracity of the effect as induced by potentially stronger manipulations. Although we did not find strong evidence for the manipulation, we did not include a control group or a present-focused frame, so our conclusions about the efficacy of the frames are limited. From our findings, it appears past- and future-focused frames are just as (in)effective as each other: it could be the case that presenting either version of the image pairs lifts pro-environmentalism, or that they both decrease it.

## Conclusions

We started this project with the assumption that we would replicate the temporal framing effect and extend the literature by investigating the drivers of this effect. However, upon failing to replicate the effect, our program of research pivoted to instead assess the possible reasons for this non-replication in a large-scale preregistered second study. Considering the results of both studies, we found little evidence that conservatives respond more favourably to environmental comparisons grounded in the past relative to the future, though they do appear to be more certain about these messages, which may backfire to *reduce* liberals’ certainty about climate change. Further work is needed to clarify the reliability of the temporal framing effect before clarifying the mechanism behind any increase in environmentalism.

## Supporting information

S1 Fig Association between political orientation and certainty that environmental changes have happened (past condition, represented in blue) or will happen (future condition, red) temporal framing condition. Solid vertical line represents the Johnson-Neyman value. To the right of this, the differences in certainty ratings by condition are significant.
(DOCX)

S2 Fig Association between anti-egalitarianism and mitigation support by temporal framing condition (past = blue, future = red). Solid vertical line represents the Johnson-Neyman value. To the right of this, the differences in certainty ratings by condition are significant.
(DOCX)

S3 Fig Association between anti-egalitarianism and adaptation support by temporal framing condition (past = blue, future = red). Solid vertical line represents the Johnson-Neyman value. To the right of this, the differences in certainty ratings by condition are significant.
(DOCX)

S4 Fig Association between SDO-D and willingness to make sacrifices for the environment by temporal framing condition (past = blue, future = red). Solid vertical lines represents the Johnson-Neyman value. Outside of these bounds, the differences in certainty ratings by condition are significant.
(DOCX)

S1 Table Model coefficients from the moderated mediation model testing the extent certainty of the environmental changes mediate the temporal framing interaction on certainty climate change is happening. (DOCX)

S2 Table Standardized regression coefficients regressing each DV on SDO-D, condition, and the interaction term. (DOCX)

S3 Table Standardized regression coefficients regressing each DV on political orientation, condition, and the interaction term for those who rated the extent images depicted effects of climate change (N = 557). (DOCX)

S4 Table Standardized regression coefficients regressing each DV on political orientation, condition, and the interaction term for those who rated the likely causes of the images (N = 545). (DOCX)

S5 Table Standardized regression coefficients regressing each explanatory variable on political orientation, condition, and the interaction term all participants, independent of rating condition. (DOCX)

S6 Table Standardized regression coefficients regressing each DV on SDO-E, condition, and the interaction term for all participants, independent of rating condition. (DOCX)

S7 Table Standardized regression coefficients regressing each DV on SDO-D, condition, and the interaction term for all participants, independent of rating condition. (DOCX)

S8 Table Standardized regression coefficients regressing each DV on authoritarian aggression, condition, and the interaction term for all participants, independent of rating condition. (DOCX)

S9 Table Standardized regression coefficients regressing each DV on authoritarian submission, condition, and the interaction term for all participants, independent of rating condition. (DOCX)

S10 Table Standardized regression coefficients regressing each DV on traditionalism, condition, and the interaction term for all participants, independent of rating condition. (DOCX)

S11 Table Standardized regression coefficients regressing each DV on the dimensions of SDO and RWA. (DOCX)

S1 Appendix (DOCX)

## References

[1] Hornsey MJ, Harris EA, Bain PG, Fielding KS. Meta-analyses of the determinants and outcomes of belief in climate change. Nat Clim Chang. 2016;6(6):622–6.

[2] Hornsey MJ, Harris EA, Fielding KS. Relationships among conspiratorial beliefs, conservatism and climate scepticism across nations. Nat Clim Chang. 2018;8: 614–620.

[3] McCright AM, Dunlap RE, Marquart-Pyatt ST. Political ideology and views about climate change in the European Union. Env Polit. 2016;25: 338–358.

[4] McCright AM, Dunlap RE. Defeating Kyoto: The conservative movement’s impact on US climate change policy. Social problems. 2003 8 1;50(3):348–73.

[5] Holden E. Trump begins year-long process to formally exit Paris climate agreement. The Guardian. 2019 Nov 5. https://www.theguardian.com/us-news/2019/nov/04/donald-trump-climate-crisis-exit-paris-agreement

[6] Saad A. Pathways of Harm: The Consequences of Trump’s Withdrawal from the Paris Climate Agreement. Environ Justice. 2018;11: 47–51.

[7] Delicado A. Environmental education technologies in a social void: The case of ‘Greendrive’. Environ Educ Res. 2012;18: 831.

[8] Owens S, Driffill L. How to change attitudes and behaviours in the context of energy. Energ Policy. 2008;36: 4412–4418.

[9] Baldwin M, Lammers J. Past-focused environmental comparisons promote proenvironmental outcomes for conservatives. PNAS. 2016;113(52):14953–7. 10.1073/pnas.1610834113 27956619 PMC5206530

[10] Jessani Z, Harris PB. Personality, politics, and denial: Tolerance of ambiguity, political orientation and disbelief in climate change. Pers Individ Dif. 2018;131: 121–123.

[11] Poortinga W, Whitmarsh L, Steg L, Böhm G, Fisher S. Climate change perceptions and their individual-level determinants: A cross-European analysis. Global Environ Chang. 2019;55: 25–35.

[12] Barnett MD, Archuleta WP, Cantu C. Politics, concern for future generations, and the environment: Generativity mediates political conservatism and environmental attitudes. J Appl Soc Psychol. 2019; 49(10):647–54.

[13] Bateman TS, O’Connor K. Felt responsibility and climate engagement: Distinguishing adaptation from mitigation. Global Environ Chang. 2016;41:206–15.

[14] McCright AM, Dunlap RE. Challenging global warming as a social problem: An analysis of the conservative movement’s counter-claims. Social problems. 2000;47:499–522.

[15] Jacques PJ, Dunlap RE, Freeman M. The organisation of denial: Conservative think tanks and environmental scepticism. Env Polit. 2008;17: 349–385.

[16] Oreskes N, Conway EM. Merchants of doubt: How a handful of scientists obscured the truth on issues from tobacco smoke to global warming. Bloomsbury Publishing USA; 2011 5 31.

[17] Brulle RJ. Institutionalizing delay: foundation funding and the creation of US climate change counter-movement organizations. Clim Change. 2014;122: 681–694.

[18] Oreskes N, Conway EM. Defeating the merchants of doubt. Nature. 2010 6;465(7299):686–7. 10.1038/465686a 20535183

[19] Feinberg M, Willer R. The moral roots of environmental attitudes. Psychol Sci. 2013;24:56–62. 10.1177/0956797612449177 23228937

[20] Klas A, Clarke EJR. The Role of Psychological Variables in Developing Effective Climate Change Message Frames In Holmes D. C. & Richardson L. M. (Eds.), Research Handbook on Communicating Climate Change. Elgar Handbooks in Energy, the Environment and Climate Change series. Cheltenham, UK: Edward Elgar Publishing; 2020.

[21] Hart PS, Nisbet EC. Boomerang effects in science communication: How motivated reasoning and identity cues amplify opinion polarization about climate mitigation policies. Communication research. 2012;39:701–23.

[22] Schuldt JP, Konrath SH, Schwarz N. “Global warming” or “climate change”? Whether the planet is warming depends on question wording. Public Opin Quart. 2011;75:115–24.

[23] Soutter ARB, Mõttus R. “Global warming” versus “climate change”: A replication on the association between political self-identification, question wording, and environmental beliefs. J Environ Psychol. 2020;101413.

[24] Caprara G, Vecchione M, Schwartz SH. Mediational role of values in linking personality traits to political orientation. Asian J Soc Psychol. 2009;12: 82–94.

[25] Van Leeuwen F, Park JH. Perceptions of social dangers, moral foundations, and political orientation. Pers Individ Dif. 2009;47: 169–173.

[26] Feygina I, Jost JT, Goldsmith RE. System justification, the denial of global warming, and the possibility of “system-sanctioned change”. Pers Social Psychol Bull. 2010;36:326–38. 10.1177/0146167209351435 20008965

[27] Jost JT, Glaser J, Kruglanski AW, Sulloway FJ. Political conservatism as motivated social cognition. Psychol Bull. 2003;129: 339–375. 10.1037/0033-2909.129.3.339 12784934

[28] Harring N, Sohlberg J. The varying effects of left—right ideology on support for the environment: Evidence from a Swedish survey experiment. Environ Pol. 2017;26:278–300.

[29] Clayton S, Koehn A, Grover E. Making sense of the senseless: Identity, justice, and the framing of environmental crises. Soc Just Res. 2013;26:301–19.

[30] Myers TA, Nisbet MC, Maibach EW, Leiserowitz AA. A public health frame arouses hopeful emotions about climate change. Climatic Change. 2012;113:1105–12.

[31] McCright AM, Charters M, Dentzman K, Dietz T. Examining the effectiveness of climate change frames in the face of a climate change denial counter‐frame. Top Cogn Sci. 2016;8: 76–97. 10.1111/tops.12171 26621098

[32] Graham J, Haidt J, Nosek BA. Liberals and conservatives rely on different sets of moral foundations. J Pers Soc Psychol. 2009;96: 1029 10.1037/a0015141 19379034

[33] Haidt J, Graham J. When morality opposes justice: Conservatives have moral intuitions that liberals may not recognize. Social Justice Research. 2007;20: 98–116.

[34] Frimer JA. Do liberals and conservatives use different moral languages? Two replications and six extensions of Graham, Haidt, and Nosek’s (2009) moral text analysis. J Res Pers. 2020; 103906.

[35] Kidwell B, Farmer A, Hardesty DM. Getting liberals and conservatives to go green: Political ideology and congruent appeals. J Consumer Res. 2013;40:350–67.

[36] Wolsko C, Ariceaga H, Seiden J. Red, white, and blue enough to be green: Effects of moral framing on climate change attitudes and conservation behaviors. J Exp Soc Psychol. 2016;65:7–19.

[37] Inbar Y, Pizarro D, Iyer R, Haidt J. Disgust sensitivity, political conservatism, and voting. Soc Psychol Pers Sci. 2012;3: 537–544.

[38] Levine AS, Kline R. A new approach for evaluating climate change communication. Climatic Change. 2017;142:301–9.

[39] Maibach EW, Nisbet M, Baldwin P, Akerlof K, Diao G. Reframing climate change as a public health issue: an exploratory study of public reactions. BMC public health. 2010;10:299 10.1186/1471-2458-10-299 20515503 PMC2898822

[40] Petrovic N, Madrigano J, Zaval L. Motivating mitigation: when health matters more than climate change. Climatic Change. 2014;126:245–54.

[41] Feldman L, Hart P. Broadening exposure to climate change news? How framing and political orientation interact to influence selective exposure. Journal of Communication. 2018;68:503–24.

[42] Lammers J, Baldwin M. Make America gracious again: Collective nostalgia can increase and decrease support for right‐wing populist rhetoric. Eur J Soc Psychol. 2020.

[43] Zimbardo PG, Boyd JN. Putting time in perspective: A valid, reliable individual-differences metric In Time perspective theory; review, research and application (pp. 17–55). Springer, 2015.

[44] Brito PQ, Vale VT. Toward an Integrated Model of Visitor’s Food Nostalgia and Gender Difference: A Festival Context. Event Management. 2018;22: 609–628.

[45] Milfont TL, Wilson J, Diniz P. Time perspective and environmental engagement: A meta‐analysis. Int J Psychol. 2012;47: 325–334. 10.1080/00207594.2011.647029 22452746

[46] Webster DM, Kruglanski AW. Individual differences in need for cognitive closure. J Pers Soc Psychol. 1994;67: 1049–1062. 10.1037//0022-3514.67.6.1049 7815301

[47] Spence A, Poortinga W, Pidgeon N. The psychological distance of climate change. Risk Analysis: An International Journal. 2012;32: 957–972. 10.1111/j.1539-6924.2011.01695.x 21992607

[48] Wang S, Hurlstone MJ, Leviston Z, Walker I, Lawrence C. Climate change from a distance: An analysis of construal level and psychological distance from climate change. Frontiers in Psychology. 2019;10:230 10.3389/fpsyg.2019.00230 30853924 PMC6395381

[49] Dunlap RE. The new environmental paradigm scale: From marginality to worldwide use. J Environ Educ. 2008;40: 3–18.

[50] Bernstein J, Szuster BW. The new environmental paradigm scale: Reassessing the operationalization of contemporary environmentalism. J Environ Educ. 2019;50: 73–83.

[51] Ellis C, Stimson JA. Ideology in America: Cambridge University Press; 2012.

[52] Feldman S, Johnston C. Understanding the determinants of political ideology: Implications of structural complexity. Pol Psychol; 2013.

[53] Klar S. A Multidimensional Study of Ideological Preferences and Priorities among the American Public. Public Opin Q. 2014;78: 344–359.

[54] Treier S, Hillygus DS. The Nature of Political Ideology in the Contemporary Electorate. Public Opin Q. 2009;73: 679–703. 10.1093/poq/nfp067

[55] Häkkinen K, Akrami N. Ideology and climate change denial. Pers Individ Dif. 2014;70: 62–65.

[56] Sidanius J, Pratto F. Social Dominance: An Intergroup Theory of Social Hierarchy and Oppression. Cambridge: Cambridge University Press 1999.

[57] Altemeyer B. The Authoritarian Specter. Harvard University Press; 1996.

[58] Stanley SK, Wilson MS. Meta-analysing the association between social dominance orientation, authoritarianism, and attitudes on the environment and climate change. J Environ Psychol. 2019;61:46–56.

[59] Stanley SK, Milfont TL, Wilson MS, Sibley CG. The influence of social dominance orientation and right-wing authoritarianism on environmentalism: A five-year cross-lagged analysis. PLOS ONE. 2019;14:e0219067 10.1371/journal.pone.0219067 31291300 PMC6619689

[60] Stanley SK, Wilson MS, Milfont TL. Exploring short-term longitudinal effects of right-wing authoritarianism and social dominance orientation on environmentalism. Pers Individ Diff. 2017;108:174–7.

[61] Ho AK, Sidanius J, Kteily N, Sheehy-Skeffington J, Pratto F, Henkel KE, et al The nature of social dominance orientation: Theorizing and measuring preferences for intergroup inequality using the new SDO₇ scale. J Pers Soc Psychol. 2015;109:1003 10.1037/pspi0000033 26479362

[62] Clarke EJ, Ling M, Kothe EJ, Klas A, Richardson B. Mitigation system threat partially mediates the effects of right‐wing ideologies on climate change beliefs. J Applied Soc Psychol. 2019;49:349–60.

[63] Stanley SK, Wilson MS, Sibley CG, Milfont TL. Dimensions of social dominance and their associations with environmentalism. Pers Individ Diff. 2017;107:228–36.

[64] Reese G. When authoritarians protect the earth—Authoritarian submission and proenvironmental beliefs: A pilot study in Germany. Ecopsychology. 2012;4:232–236.

[65] Zhang JW, Howell RT, Bowerman T. Validating a brief measure of the Zimbardo Time Perspective Inventory. Time & Society. 2013;22:391–409.

[66] Heimberg LK. The measurement of future time perspective (Doctoral dissertation). 1963.

[67] Lyu H, Huang X. Development and validation of future time perspective scale for adolescents and young adults. Time & Society. 2016;25:533–51.

[68] Mello ZR, Worrell FC. The adolescent time inventory-English. Berkeley: The University of California 2007.

[69] Shell DF, Husman J. The multivariate dimensionality of personal control and future time perspective beliefs in achievement and self-regulation. Contemporary educational psychology. 2001;26:481–506. 10.1006/ceps.2000.1073 11681829

[70] Baldwin M, White MH, Sullivan D. Nostalgia for America’s past can buffer collective guilt. Eur J Soc Psychol. 2018;48:433–46.

[71] Liu JH, Sibley CG. Hope for the future? Understanding self-sacrifice in the face of global warming among young citizens of the world. ASAP. 2012;12:190–203.

[72] Long JA. Interactions: Comprehensive, User-Friendly Toolkit for Probing Interactions. R package version 1.1.0. 2019; Accessed from https://cran.r-project.org/package=interactions.

[73] Hayes AF. Introduction to mediation, moderation, and conditional process analysis: A regression-based approach. Guilford publications; 2017 12 13.

[74] Duckitt J, Bizumic B, Krauss SW, Heled E. A tripartite approach to right-wing authoritarianism: The Authoritarianism-Conservatism-Traditionalism model. Polit Psychol. 2010; 31, 685–715.

[75] Loy LS, Spence A. Reducing, and bridging, the psychological distance of climate change. Journal of Environmental Psychology. 2020;67:101388.

[76] Dunlap RE, Van Liere KD, Mertig AG, Jones RE. New trends in measuring environmental attitudes: measuring endorsement of the new ecological paradigm: a revised NEP scale. J Social Issues. 2000;56:425–42.

[77] Nisbet MC. Communicating climate change: Why frames matter for public engagement. Environment: Science and policy for sustainable development. 2009;51:12–23.

[78] Nisbet EC, Hart PS, Myers T, Ellithorpe M. Attitude change in competitive framing environments? Open-/closed-mindedness, framing effects, and climate change. J Communication. 2013;63:766–85.

[79] Makel MC, Plucker JA, Hegarty B. Replications in psychology research: How often do they really occur? Perspectives Psychol Sci. 2012;7:537–42. 10.1177/1745691612460688 26168110

[80] Martin GN, Clarke RM. Are psychology journals anti-replication? A snapshot of editorial practices. Frontiers in Psychology. 2017;8:523 10.3389/fpsyg.2017.00523 28443044 PMC5387793

[81] Everett JAC, Earp BD. A tragedy of the (academic) commons: interpreting the replication crisis in psychology as a social dilemma for early-career researchers. Front Psychol. 2015;6: 1152 10.3389/fpsyg.2015.01152 26300832 PMC4527093

[82] Lilienfeld SO. Psychology’s replication crisis and the grant culture: Righting the ship. Perspectives on Psychological Science. 2017;12: 660–664. 10.1177/1745691616687745 28727961

[83] Fanelli D. “Positive” results increase down the hierarchy of the sciences. PlOS ONE. 2010;5:e10068 10.1371/journal.pone.0010068 20383332 PMC2850928

[84] Open SC. Psychology. Estimating the reproducibility of psychological science. Science. 2015;349(6251):aac4716 10.1126/science.aac4716 26315443

[85] Anvari F, Lakens D. The replicability crisis and public trust in psychological science. Compr Results Soc Psychol. 2018;3: 266–286.

[86] Wingen T, Berkessel JB, Englich B. No Replication, No Trust? How Low Replicability Influences Trust in Psychology. Soc Psychol Pers Sci. 2019; 10.1177/1948550619877412

[87] Button KS, Ioannidis JP, Mokrysz C, Nosek BA, Flint J, Robinson ES, et al Power failure: why small sample size undermines the reliability of neuroscience. Nature reviews neuroscience. 2013;14:365–76. 10.1038/nrn3475 23571845

[88] Blake KR, Gangestad S. On Attenuated Interactions, Measurement Error, and Statistical Power: Guidelines for Social and Personality Psychologists. Pers Soc Psychol Bull. 2020; 0146167220913363. 10.1177/0146167220913363 32208875

[89] Simonsohn U. No-way Interactions, The Winnower 7:e14255990552. 2015; 10.15200/winn.142559.90552

[90] Shrout PE, Rodgers JL. Psychology, science, and knowledge construction: Broadening perspectives from the replication crisis. Annual Review of Psychology. 2018;69:487–510. 10.1146/annurev-psych-122216-011845 29300688

[91] Kim I, Hammond MD, Milfont T L. Do Past-Focused Environmental Messages Promote Pro-Environmentalism to Conservatives? A Pre-Registered Replication, J Environ Psychol. In press; 10.1016/j.jenvp.2020.101547

[92] Maxwell SE, Lau MY, Howard GS. Is psychology suffering from a replication crisis? What does “failure to replicate” really mean? Am Psychol. 2015;70: 487–498. 10.1037/a0039400 26348332

[93] Klein RA, Ratliff KA, Vianello M, Adams RB Jr, Bahník Š, Bernstein MJ, et al Investigating variation in replicability. Soc Psychol. 2014.

[94] Rickard LN, Yang ZJ, Schuldt JP. Here and now, there and then: How “departure dates” influence climate change engagement. Global Environ Chang. 2016;38: 97–107.

[95] Roh S, McComas KA, Rickard LN, Decker DJ. How motivated reasoning and temporal frames may polarize opinions about wildlife disease risk. Sci Commun. 2015;37: 340–370.

[96] Federico CM, Malka A. The contingent, contextual nature of the relationship between needs for security and certainty and political preferences: Evidence and implications. Pol Psychol. 2018;39: 3–48.

