# Supplementary material for: The effects of a temporal framing manipulation on environmentalism: A replication and extension
Source: PLoS One. 2021 Feb 11;16(2):e0246058. doi: 10.1371/journal.pone.0246058 (PMC7877654; doi:10.1371/journal.pone.0246058)
Supplement: S1 Fig — Solid vertical line represents the Johnson-Neyman value. To the right of this, the differences in certainty ratings by condition are significant. (DOCX) [file pone.0246058.s001.docx]

*
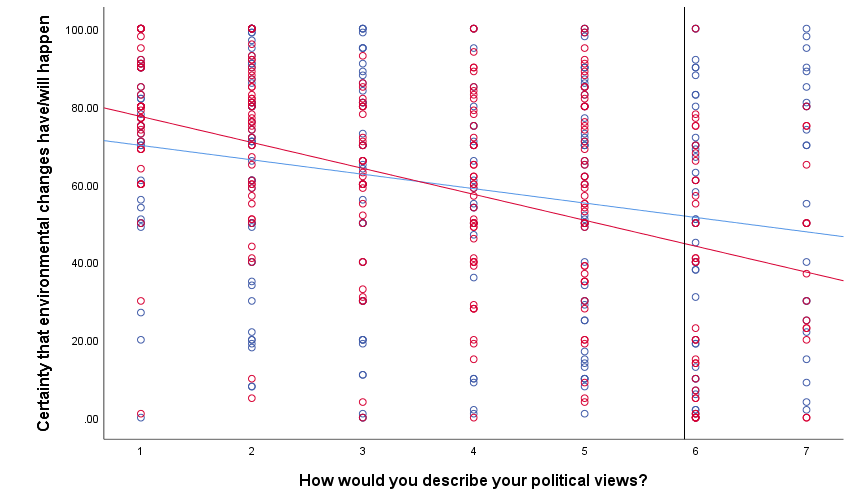
*

*Figure S1.* Association between political orientation and certainty that environmental changes have happened (past condition, represented in blue) or will happen (future condition, red) temporal framing condition.

*Note.* Solid vertical line represents the Johnson-Neyman value. To the right of this, the differences in certainty ratings by condition are significant.
