# Supplementary material for: The effects of a temporal framing manipulation on environmentalism: A replication and extension
Source: PLoS One. 2021 Feb 11;16(2):e0246058. doi: 10.1371/journal.pone.0246058 (PMC7877654; doi:10.1371/journal.pone.0246058)
Supplement: S3 Fig — Solid vertical line represents the Johnson-Neyman value. To the right of this, the differences in certainty ratings by condition are significant. (DOCX) [file pone.0246058.s003.docx]

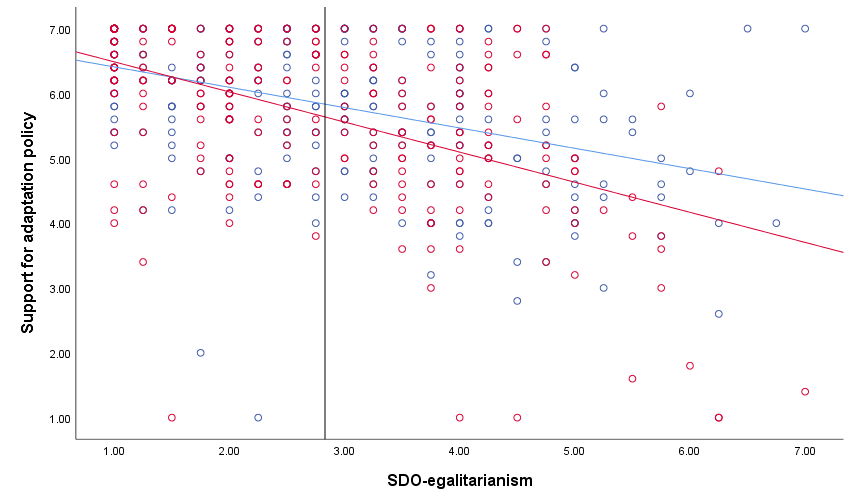


*Figure S3.* Association between anti-egalitarianism and adaptation support by temporal framing condition (past = blue, future = red).

Note. Solid vertical line represents the Johnson-Neyman value. To the right of this, the differences in certainty ratings by condition are significant.
