# Supplementary material for: The effects of a temporal framing manipulation on environmentalism: A replication and extension
Source: PLoS One. 2021 Feb 11;16(2):e0246058. doi: 10.1371/journal.pone.0246058 (PMC7877654; doi:10.1371/journal.pone.0246058)
Supplement: S4 Fig — Solid vertical lines represents the Johnson-Neyman value. Outside of these bounds, the differences in certainty ratings by condition are significant. (DOCX) [file pone.0246058.s004.docx]

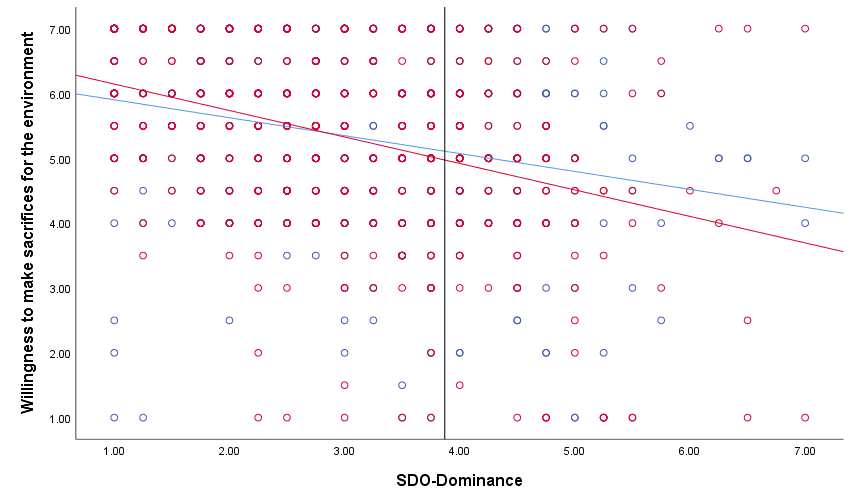


Figure S4. Association between SDO-D and willingness to make sacrifices for the environment by temporal framing condition (past = blue, future = red).

*Note.* Solid vertical lines represents the Johnson-Neyman value. Outside of these bounds, the differences in certainty ratings by condition are significant.
