# Supplementary material for: The effects of a temporal framing manipulation on environmentalism: A replication and extension
Source: PLoS One. 2021 Feb 11;16(2):e0246058. doi: 10.1371/journal.pone.0246058 (PMC7877654; doi:10.1371/journal.pone.0246058)
Supplement: S1 Table — (DOCX) [file pone.0246058.s005.docx]

Table S1.

Model coefficients from the moderated mediation model testing the extent certainty of the environmental changes mediate the temporal framing interaction on certainty climate change is happening.

|  | Certainty changes will/have happened (M) | | | Climate change certainty (Y) | | |
| --- | --- | --- | --- | --- | --- | --- |
|  | Coefficient | Standard error | *p* | Coefficient | Standard error | *p* |
| Political ideology (X) | -0.739 | 1.952 | .705 | -3.878 | 1.562 | .013 |
| Certainty changes will/have happened (M) | -- | -- | -- | 0.392 | 0.035 | < .001 |
| Temporal framing condition (W) | 10.406 | 5.219 | .047 | 1.657 | 4.190 | .693 |
| Political ideology X temporal frame | -2.965 | 1.255 | .019 | -1.751 | 1.009 | .083 |
|  | R^2^ = .119  F(3, 531) = 23.98, *p* < .001 | | | R^2^ = .446  F(4, 530) = 106.68, *p* < .001 | | |
