# Supplementary material for: The effects of a temporal framing manipulation on environmentalism: A replication and extension
Source: PLoS One. 2021 Feb 11;16(2):e0246058. doi: 10.1371/journal.pone.0246058 (PMC7877654; doi:10.1371/journal.pone.0246058)
Supplement: S2 Table — (DOCX) [file pone.0246058.s006.docx]

Table S2.

|  | Climate change belief | Climate change certainty | Climate change causes | Willing to sacrifice | Support mitigation | Support adaptation |
| --- | --- | --- | --- | --- | --- | --- |
| **Step 1** | R^2^ = .189***** | R^2^ = .141***** | R^2^ = .180***** | R^2^ = .130***** | R^2^ = .190***** | R^2^ = .138***** |
| SDO-D | -.433*** | -.367*** | .424*** | -.357*** | -.434*** | -.367*** |
| Condition | -.039 | -.078 | -.021 | -.057 | -.044 | -.059 |
| **Step 2** | ΔR^2^ = .000 | ΔR^2^ = .000 | ΔR^2^ = .000 | ΔR^2^ = .001 | ΔR^2^ = .000 | ΔR^2^ = .001 |
| SDO-D | -.432*** | -.335** | .468*** | -.266* | -.398** | -.274* |
| Condition | .038 | -.056 | .010 | .007 | -.019 | .007 |
| SDO-D X condition | -.001 | -.041 | -.056 | -.115 | -.046 | -.118 |

*Standardized regression coefficients regressing each DV on SDO-D, condition, and the interaction term.*
