# Supplementary material for: The effects of a temporal framing manipulation on environmentalism: A replication and extension
Source: PLoS One. 2021 Feb 11;16(2):e0246058. doi: 10.1371/journal.pone.0246058 (PMC7877654; doi:10.1371/journal.pone.0246058)
Supplement: S3 Table — (DOCX) [file pone.0246058.s007.docx]

Table S3. *Standardized regression coefficients regressing each DV on political orientation, condition, and the interaction term for those who rated the extent images depicted effects of climate change (N = 557).*

|  | Pro-environmental attitudes | Climate change belief | Climate change certainty | Climate change causes | Willingness to sacrifice | Support for mitigation policy | Support for adaptation policy |
| --- | --- | --- | --- | --- | --- | --- | --- |
| **Step 1** | R^2^ = .031** | R^2^ = .126*** | R^2^ = .098*** | R^2^ = .097*** | R^2^ = .120*** | R^2^ = .083*** | R^2^ = .008 |
| Political orientation | -.168*** | -.353*** | -.317*** | .308*** | -.345*** | -.287*** | -.066 |
| Condition | -.048 | .049 | -.018 | -.055 | -.009 | .000 | -.058 |
| **Step 2** | ΔR^2^ = .004 | ΔR^2^ = .005 | ΔR^2^ = .003 | ΔR^2^ = .003 | ΔR^2^ = .004 | ΔR^2^ = .004 | ΔR^2^ = .000 |
| Political orientation | .037 | -.142 | -.152 | .128 | -.162 | -.089 | -.058 |
| Condition | .088 | .190* | .091 | -.175 | .113 | .132 | -.052 |
| Political orientation X condition | -.260 | -.269 | -.209 | .228 | -.233 | -.251 | -.011 |

*Note. *** p* < .001, *** p* < .01*, * p* < .05
