# Supplementary material for: The effects of a temporal framing manipulation on environmentalism: A replication and extension
Source: PLoS One. 2021 Feb 11;16(2):e0246058. doi: 10.1371/journal.pone.0246058 (PMC7877654; doi:10.1371/journal.pone.0246058)
Supplement: S4 Table — (DOCX) [file pone.0246058.s008.docx]

Table S4. *Standardized regression coefficients regressing each DV on political orientation, condition, and the interaction term for those who rated the likely causes of the images (N = 545).*

|  | Pro-environmental attitudes | Climate change belief | Climate change certainty | Climate change causes | Willingness to sacrifice | Support for mitigation policy | Support for adaptation policy |
| --- | --- | --- | --- | --- | --- | --- | --- |
| **Step 1** | R^2^ = .020** | R^2^ = .131*** | R^2^ = .080*** | R^2^ = .079*** | R^2^ = .073*** | R^2^ = .055*** | R^2^ = .011* |
| Political orientation | -.136** | -.360*** | -.283*** | .279*** | -.271*** | -.226*** | -.009 |
| Condition | .042 | .041 | .027 | -.045 | .024 | .069 | .106* |
| **Step 2** | ΔR^2^ = .000 | ΔR^2^ = .000 | ΔR^2^ = .005 | ΔR^2^ = .004 | ΔR^2^ = .000 | ΔR^2^ = .000 | ΔR^2^ = .000 |
| Political orientation | -.143 | -.417** | -.064 | .466*** | -.241 | -.217 | -.018 |
| Condition | .037 | .001 | .183 | .088 | .045 | .075 | .100 |
| Political orientation X condition | .009 | .071 | -.282 | -.239 | -.038 | -.011 | .012 |

*Note. *** p* < .001, *** p* < .01*, * p* < .05
