# Supplementary material for: The effects of a temporal framing manipulation on environmentalism: A replication and extension
Source: PLoS One. 2021 Feb 11;16(2):e0246058. doi: 10.1371/journal.pone.0246058 (PMC7877654; doi:10.1371/journal.pone.0246058)
Supplement: S5 Table — (DOCX) [file pone.0246058.s009.docx]

Table S5. *Standardized regression coefficients regressing each explanatory variable on political orientation, condition, and the interaction term all participants, independent of rating condition.*

|  | Certainty changes had/will happen | Temporal distance | Nostalgia | Past positive time perspective |
| --- | --- | --- | --- | --- |
| **Step 1** | R^2^ = .041*** | R^2^ = .009** | R^2^ = .004 | R^2^ = .040*** |
| Political orientation | -.202*** | -.093** | -.007 | .199*** |
| Condition | -.012 | -.014 | -.066* | -.018 |
| **Step 2** | ΔR^2^ = .001 | ΔR^2^ = .000 | ΔR^2^ = .001 | ΔR^2^ = .002 |
| Political orientation | -.094 | -.097 | -.092 | .351*** |
| Condition | .062 | -.017 | -.124 | .086 |
| Political orientation X condition | -.137 | .006 | .109 | -.194 |

*Note. *** p* < .001, *** p* < .01*, * p* < .05
