# Supplementary material for: The effects of a temporal framing manipulation on environmentalism: A replication and extension
Source: PLoS One. 2021 Feb 11;16(2):e0246058. doi: 10.1371/journal.pone.0246058 (PMC7877654; doi:10.1371/journal.pone.0246058)
Supplement: S6 Table — (DOCX) [file pone.0246058.s010.docx]

Table S6. *Standardized regression coefficients regressing each DV on SDO-E, condition, and the interaction term for all participants, independent of rating condition.*

|  | Pro-environmental attitudes | Climate change belief | Climate change certainty | Climate change causes | Willingness to sacrifice | Support for mitigation policy | Support for adaptation policy |
| --- | --- | --- | --- | --- | --- | --- | --- |
| **Step 1** | R^2^ = .044*** | R^2^ = .136*** | R^2^ = .092*** | R^2^ = .078*** | R^2^ = .111*** | R^2^ = .119*** | R^2^ = .030*** |
| SDO-E | -.211*** | -.368*** | -.302*** | .276*** | -.333*** | -.344*** | -.173*** |
| Condition | -.012 | .027 | -.013 | -.035 | -.009 | .020 | .019 |
| **Step 2** | ΔR^2^ = .001 | ΔR^2^ = .000 | ΔR^2^ = .002 | ΔR^2^ = .000 | ΔR^2^ = .001 | ΔR^2^ = .000 | ΔR^2^ = .000 |
| SDO-E | -.110 | -.323*** | -.156 | .327*** | -.235** | -.346*** | -.114 |
| Condition | .071 | .064 | .109 | .008 | .071 | .018 | .068 |
| SDO-E X condition | -.134 | -.059 | -.195 | -.069 | -.130 | .002 | -.078 |

*Note. *** p* < .001, *** p* < .01*, * p* < .05
