# Supplementary material for: The effects of a temporal framing manipulation on environmentalism: A replication and extension
Source: PLoS One. 2021 Feb 11;16(2):e0246058. doi: 10.1371/journal.pone.0246058 (PMC7877654; doi:10.1371/journal.pone.0246058)
Supplement: S7 Table — (DOCX) [file pone.0246058.s011.docx]

Table S7. *Standardized regression coefficients regressing each DV on SDO-D, condition, and the interaction term for all participants, independent of rating condition.*

|  | Pro-environmental attitudes | Climate change belief | Climate change certainty | Climate change causes | Willingness to sacrifice | Support for mitigation policy | Support for adaptation policy |
| --- | --- | --- | --- | --- | --- | --- | --- |
| **Step 1** | R^2^ = .045*** | R^2^ = .090*** | R^2^ = .063*** | R^2^ = .068*** | R^2^ = .098*** | R^2^ = .098*** | R^2^ = .035*** |
| SDO-D | -.213*** | -.298*** | -.251*** | .257*** | -.313*** | -.312*** | -.186*** |
| Condition | -.014 | .027 | -.012 | -.035 | -.010 | .020 | .018 |
| **Step 2** | ΔR^2^ = .003 | ΔR^2^ = .002 | ΔR^2^ = .008** | ΔR^2^ = .000 | ΔR^2^ = .004* | ΔR^2^ = .000 | ΔR^2^ = .000 |
| SDO-D | -.040 | -.178 | .021 | .264** | -.131 | -.279** | -.196* |
| Condition | .127 | .124 | .210** | -.030 | .139 | .046 | .010 |
| SDO-D X condition | -.228 | -.158 | -.360** | -.008 | -.240* | -.043 | .014 |

*Note. *** p* < .001, *** p* < .01*, * p* < .05
