# Supplementary material for: The effects of a temporal framing manipulation on environmentalism: A replication and extension
Source: PLoS One. 2021 Feb 11;16(2):e0246058. doi: 10.1371/journal.pone.0246058 (PMC7877654; doi:10.1371/journal.pone.0246058)
Supplement: S8 Table — (DOCX) [file pone.0246058.s012.docx]

Table S7. *Standardized regression coefficients regressing each DV on Authoritarian Aggression, condition, and the interaction term for all participants, independent of rating condition.*

|  | Pro-environmental attitudes | Climate change belief | Climate change certainty | Climate change causes | Willingness to sacrifice | Support for mitigation policy | Support for adaptation policy |
| --- | --- | --- | --- | --- | --- | --- | --- |
| **Step 1** | R^2^ = .004 | R^2^ = .061*** | R^2^ = .050*** | R^2^ = .043*** | R^2^ = .049*** | R^2^ = .028*** | R^2^ = .000 |
| Authoritarian aggression | -.058 | -.244*** | -.224*** | .203*** | -.221*** | -.167*** | -.006 |
| Condition | -.011 | .027 | -.014 | -.034 | -.010 | .020 | .021 |
| **Step 2** | ΔR^2^ = .000 | ΔR^2^ = .000 | ΔR^2^ = .002 | ΔR^2^ = .002 | ΔR^2^ = .000 | ΔR^2^ = .000 | ΔR^2^ = .000 |
| Authoritarian aggression | -.079 | -.237* | -.094 | .340*** | -.221* | -.145 | .010 |
| Condition | -.035 | .035 | .141 | .131 | -.010 | .047 | .041 |
| Authoritarian aggression X condition | .031 | -.011 | -.204 | -.216 | .000 | -.035 | -.026 |

*Note. *** p* < .001, *** p* < .01*, * p* < .05
