# Supplementary material for: The effects of a temporal framing manipulation on environmentalism: A replication and extension
Source: PLoS One. 2021 Feb 11;16(2):e0246058. doi: 10.1371/journal.pone.0246058 (PMC7877654; doi:10.1371/journal.pone.0246058)
Supplement: S9 Table — (DOCX) [file pone.0246058.s013.docx]

Table S8. *Standardized regression coefficients regressing each DV on Authoritarian Submission, condition, and the interaction term for all participants, independent of rating condition.*

|  | Pro-environmental attitudes | Climate change belief | Climate change certainty | Climate change causes | Willingness to sacrifice | Support for mitigation policy | Support for adaptation policy |
| --- | --- | --- | --- | --- | --- | --- | --- |
| **Step 1** | R^2^ = .013** | R^2^ = .062*** | R^2^ = .052*** | R^2^ = .057*** | R^2^ = .045*** | R^2^ = .045*** | R^2^ = .003 |
| Submission | -.112*** | -.247*** | -.227*** | .236*** | -.212*** | -.211*** | -.046 |
| Condition | -.014 | .024 | -.017 | -.031 | -.012 | .017 | .020 |
| **Step 2** | ΔR^2^ = .001 | ΔR^2^ = .000 | ΔR^2^ = .001 | ΔR^2^ = .003 | ΔR^2^ = .001 | ΔR^2^ = .000 | ΔR^2^ = .000 |
| Submission | -.205* | -.292** | -.141 | .388*** | -.289** | -.253** | -.064 |
| Condition | -.104 | -.020 | .067 | .117 | -.088 | -.024 | .002 |
| Submission X condition | .132 | .063 | -.121 | -.214 | .110 | .058 | .026 |

*Note. *** p* < .001, *** p* < .01*, * p* < .05
