# Supplementary material for: The effects of a temporal framing manipulation on environmentalism: A replication and extension
Source: PLoS One. 2021 Feb 11;16(2):e0246058. doi: 10.1371/journal.pone.0246058 (PMC7877654; doi:10.1371/journal.pone.0246058)
Supplement: S10 Table — (DOCX) [file pone.0246058.s014.docx]

Table S9. *Standardized regression coefficients regressing each DV on Traditionalism, condition, and the interaction term for all participants, independent of rating condition.*

|  | Pro-environmental attitudes | Climate change belief | Climate change certainty | Climate change causes | Willingness to sacrifice | Support for mitigation policy | Support for adaptation policy |
| --- | --- | --- | --- | --- | --- | --- | --- |
| **Step 1** | R^2^ = .065*** | R^2^ = .078*** | R^2^ = .089*** | R^2^ = .077*** | R^2^ = .050*** | R^2^ = .099*** | R^2^ = .016*** |
| Traditionalism | -.254*** | -.278*** | -.298*** | .275*** | -.223*** | -.314*** | -.126 |
| Condition | -.019 | .023 | -.020 | -.028 | -.013 | .013 | .017 |
| **Step 2** | ΔR^2^ = .000 | ΔR^2^ = .000 | ΔR^2^ = .000 | ΔR^2^ = .000 | ΔR^2^ = .000 | ΔR^2^ = .000 | ΔR^2^ = .000 |
| Traditionalism | -.262** | -.324*** | -.243** | .320*** | -.227* | -.375*** | -.170 |
| Condition | -.025 | -.017 | .029 | .011 | -.016 | -.041 | -.022 |
| Traditionalism X condition | .010 | .061 | -.075 | -.060 | .005 | .082 | .059 |

*Note. *** p* < .001, *** p* < .01*, * p* < .05
