# Supplementary material for: The effects of a temporal framing manipulation on environmentalism: A replication and extension
Source: PLoS One. 2021 Feb 11;16(2):e0246058. doi: 10.1371/journal.pone.0246058 (PMC7877654; doi:10.1371/journal.pone.0246058)
Supplement: S11 Table — (DOCX) [file pone.0246058.s015.docx]

Table S10. *Standardized regression coefficients regressing each DV on the dimensions of SDO and RWA.*

|  | Pro-environmental attitudes | Climate change belief | Climate change certainty | Climate change causes | Willingness to sacrifice | Support for mitigation policy | Support for adaptation policy |
| --- | --- | --- | --- | --- | --- | --- | --- |
|  | R^2^ = .105*** | R^2^ = .167*** | R^2^ = .138*** | R^2^ = .123*** | R^2^ = .135*** | R^2^ = .179*** | R^2^ = .061*** |
| SDO-E | -.138** | -.284*** | -.215*** | .158*** | -.203*** | -.244*** | -.118** |
| SDO-D | -.114** | -.027 | -.015 | .071 | -.123** | -.107** | -.148** |
| Authoritarian aggression | .159*** | -.004 | -.013 | -.027 | -.006 | .119** | .160*** |
| Conservatism | .018 | -.032 | -.012 | .067 | -.018 | -.019 | .019 |
| Traditionalism | -.250*** | -.164*** | -.215*** | .182*** | -.107** | -.245*** | -.119** |

*Note. *** p* < .001, *** p* < .01*, * p* < .05
