# Supplementary figures and images for: The effects of a temporal framing manipulation on environmentalism: A replication and extension
Source: PLoS One. 2021 Feb 11;16(2):e0246058. doi: 10.1371/journal.pone.0246058 (PMC7877654; doi:10.1371/journal.pone.0246058)

**Appendix.**

Petitions used for the behavioural measure:
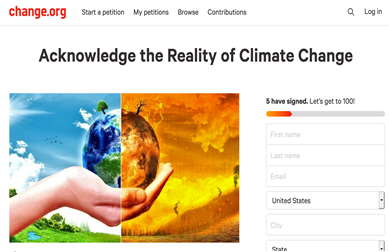

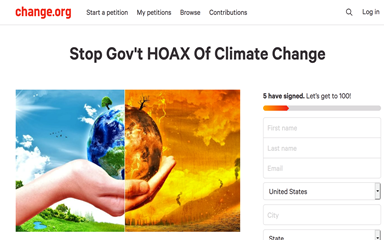

Supplement: S1 Appendix — (DOCX) [file pone.0246058.s016.docx]
